# A scoping review of therapeutic mentoring for youth mental health

**DOI:** 10.3389/frcha.2025.1509971

**Published:** 2025-01-27

**Authors:** Alexandra Werntz, Jean E. Rhodes, Hannah Brockstein, Lindsay Fallon, Amy Cook

**Affiliations:** ^1^ Center for Evidence-Based Mentoring, University of Massachusetts Boston, Boston, MA, United States; ^2^ Department of Psychology, Ohio University, Athens, OH, United States; ^3^ Counseling and School Psychology, University of Massachusetts Boston, Boston, MA, United States; ^4^ Office of the Provost and Vice Chancellor for Academic Affairs, University of Massachusetts Boston, Boston, MA, United States

**Keywords:** scoping review, youth mental health, at-risk youth, mentoring, therapeutic mentoring

## Abstract

**Introduction:**

Therapeutic mentoring, which leverages paraprofessional care, is a potential way to scale access to care to address the youth mental health crisis. This scoping review synthesizes the current state of research on self-designated therapeutic mentoring programs for youth mental health.

**Method:**

A systematic search was conducted across four databases using the term “therapeutic mento*” and related keywords, taking a label-first approach to describe the available literature. Inclusion criteria were peer-reviewed articles about research on therapeutic mentoring in the US, written in English. Data were extracted on study characteristics, intervention details, mentor background, and outcomes.

**Results:**

Eighteen empirical articles were identified, published between 2003 and 2024. Most studies focused on at-risk youth from diverse backgrounds. Therapeutic mentoring programming varied, although most (*N* = 13) studies examined the Campus Connections program. Mentors were typically trained paraprofessionals or undergraduate students supervised by clinical professionals. Only two randomized controlled trials were found, both of the Campus Connections program.

**Discussion:**

The review revealed a lack of rigorous experimental studies on therapeutic mentoring efficacy, as defined by studies that use the term therapeutic mentoring. While some studies showed promising effects, more research is needed to establish the definition of therapeutic mentoring and whether it is an acceptable and effective intervention for youth mental health.

**Conclusion:**

A clear definition of therapeutic mentoring is needed to advance the field and facilitate systematic evaluation of its effectiveness in supporting youth mental health. Future research should prioritize developing program models that align with diverse youth's cultural values, conducting randomized controlled trials, examining program components, and developing standardized measures for assessing therapeutic mentoring outcomes.

## 1 Introduction

In the U.S., between 20% and 30% of children are in need of psychological services, but only about a third of these children actually receive care (1–3). Children from communities of color, as well as those residing in under-resourced neighborhoods and communities, have the highest rates of unmet mental health needs (4, 5). Since highly-trained providers (e.g., psychologists, psychiatrists, counselors) will never meet the demand for mental health care (1, 6, 7), there is an urgent need to expand the mental health workforce to include well-trained paraprofessionals, including volunteer and paid mentors. With appropriate training and supervision, paraprofessional mentors can be leveraged to support youth mental health. This manuscript focuses on *therapeutic mentoring*, an increasingly utilized but poorly defined paraprofessional role in youth mental health services. Through a scoping review of peer-reviewed literature, we aim to synthesize existing knowledge about self-designated therapeutic mentoring programs, clarify key concepts and intervention characteristics, and identify critical gaps to guide future research and practice in this emerging field.

### 1.1 Background

There is growing evidence to suggest that paraprofessionals (i.e., providers with limited training who assist more advanced mental health professionals) can support and deliver effective, evidence-based interventions. This includes both paid and volunteer mentors who are increasingly being asked to address depression and other mental health challenges among youth (8, 9). In fact, families often turn to mentoring programs to support their youth's mental health (10, 11). Although the overall effectiveness of youth mentoring on well-being outcomes (broadly defined) is minimal when examined across all mentoring studies (12, 13), research on particular programs (14, 15) and meta-analytic evidence suggests that when mentors use targeted interventions with their mentees, they are particularly effective in moving the needle on youth outcomes (16).

Despite the promise of youth mentoring to help reduce the overall burden of mental illness, the field lacks definitions and clarity around how paraprofessional mentoring can be used to support youth mental health. In the US, mentoring is generally used as an umbrella term that can cover almost any situation in which a caring, supportive person is paired with another person seeking support outside of professional helping relationships. There is also diversity in mentoring program approaches (e.g., intergenerational, peer) and settings (schools, communities, workplaces). And, despite the growing interest in deploying paraprofessionals, to address youth mental health (17), there is a lack of clear terminology for the roles that paraprofessional mentors can play in youth's lives. In this manuscript, we focus on *therapeutic mentoring*, a paraprofessional role that has growing interest in the field, however to our understanding lacks a unifying definition.

Early descriptions of therapeutic mentoring reflect both humanistic and psychoanalytic approaches to therapy, particularly in how they emphasize the importance of personal growth, emotional connection, and the therapeutic relationship. Humanistic therapy, championed by figures like Carl Rogers and Abraham Maslow, stressed the individual's capacity for self-actualization and the critical role of empathetic, authentic relationships in fostering development (18). The therapeutic mentoring model described by Radda (19) highlights a developmental approach where mentors are personally involved, empathetic, and help adolescents find and pursue their dreams. “The mentor must be personally involved, be empathetic not sympathetic, demonstrate charisma and expertness, help the adolescent find his DREAM, encourage and give hope to that DREAM, judiciously self-disclose, model responsible behavior, demand the best from the protege and renegotiate the relationship when the adolescent demands a new maturity” (19). This description of the need for mentors to negotiate adolescents' dreams and renegotiate the relationship as maturity develops mirrors psychoanalytic ideas of navigating conflicts between dependence and independence, while the therapeutic alliance remains crucial for growth (20).

By the 2010's, therapeutic mentoring began to be professionalized, and recognized as a billable service under Medicaid in some states for youth with behavioral health needs, allowing for further professionalization of the practice. Massachusetts was one of the early leaders in recognizing therapeutic mentoring as a Medicaid-billable service. One factor that precipitated its expansion in Massachusetts was the Rosie D. v. Romney lawsuit, which was filed in 2001 on behalf of Medicaid-eligible children with emotional, behavioral, or psychiatric disabilities who lacked access to necessary home-based services. In 2006, the court ruled that Massachusetts had violated the Early and Periodic Screening, Diagnostic, and Treatment (EPSDT) provisions of the federal Medicaid Act by failing to provide required home-based services to thousands of children across the state. As a result of the ruling, Massachusetts was required to develop and implement a remedial plan to address these violations and expand access to home-based services for youth with serious emotional disturbances through its Community Health Workers (CHWs) program. The remedial plan included the development and expansion of several services, including therapeutic mentoring, as part of a comprehensive system of care for youth with mental health needs. In 2009, Massachusetts implemented a new children's behavioral health initiative that included therapeutic mentoring as one of several “hub-dependent” services that could be billed to Medicaid when prescribed as part of a youth's treatment plan. In the current version, therapeutic mentoring includes structured, one-on-one support services provided to individuals under 21-years-old across settings, including youths' homes, schools, and communities (21). These strength-based services address daily living, social, and communication needs, aligning with a behavioral health treatment plan or Individual Care Plan (ICP) and supervised by clinicians or another person on the care team. Therapeutic mentoring focuses on developing age-appropriate behaviors, interpersonal communication, problem-solving, and social interactions with peers and adults. The goal is to ensure youth success in various social contexts, help them learn new skills, and make functional progress. Mentors supervise interactions and engage youth in discussions about effective strategies for peer interactions, with progress documented and reported regularly to the youth's current treatment providers. To be eligible, these services must be necessary to achieve established goals in an existing treatment plan or ICP and show documented progress toward meeting these goals.

Across the U.S. there are additional programs that offer therapeutic mentoring services with slightly different descriptions and training requirements. For example, in Alabama's Medicaid-eligible rehabilitative service supervised therapeutic mentors (who can have a wide range of certifications, including qualified mental health provider—non-degreed or a licensed registered nursing degree) work under an individual's treatment plan (22). Likewise, Illinois' Pathways to Success program for Medicaid-enrolled children incorporates therapeutic mentoring services and Connecticut's therapeutic support staff provide therapeutic mentoring through the Department of Children and Families (23). There are also examples of self-designated therapeutic mentoring at the program level, such as the YMCA's Reach & Rise program (24), as well as private and community-based agencies in Virginia (25) and Maryland.

Campus Connections (originally Campus Corps), is another example of a self-designated therapeutic youth mentoring program. This program trains undergraduate students to serve as mentors, incorporating mental health professionals to address participants' needs (26). Established in 2009 at Colorado State University, CC was developed in response to community needs for enhanced services for at-risk youth (26). The program's distinctive approach includes organizing mentor-mentee pairs into small groups called “mentor families”, providing exposure to a college campus environment, offering academic support and pro-social activities, and integrating mental health services delivered by licensed therapists (27, 28).

A major challenge to understanding the therapeutic mentoring landscape is that there does not appear to be an accepted definition of what constitutes this approach. This has led to confusion and inconsistency across the field wherein some programs self-designate as therapeutic mentoring while others who may be engaging in its core practices do not. For example, the Fostering Healthy Futures Program (29), which does not designate itself as therapeutic mentoring, deploys supervised social work students who identify and address mental health issues. This inconsistent use of the therapeutic mentoring title and the lack of an accepted definition has led to challenges in characterizing the field and examining whether self-designated *therapeutic mentoring* is an effective way of supporting youth mental health. And, without a clear term for therapeutic mentoring that explicitly targets youth mental health, the field cannot systematically study the outcomes. To advance the field and maximize its impact, it is crucial to take stock of the field and then to develop a clear, comprehensive definition of therapeutic mentoring. Such a definition would facilitate more systematic study of the practice, enhance its visibility within the broader mental health landscape, and delineate the specific roles and limitations of therapeutic mentors. By establishing these boundaries, practitioners, researchers, and policymakers can better understand, implement, and evaluate therapeutic mentoring programs, ultimately improving their efficacy in supporting youth mental health outcomes.

### 1.2 Current study

To understand how *therapeutic mentoring* is currently used in academic discourse and peer-reviewed publications, an exploratory scoping review is necessary to describe the research landscape. To our knowledge, this is the first review of existing literature on *therapeutic mentoring,* thus we have opted to take a label-first approach (in contrast to a definition-first approach where the theorized core features of therapeutic mentoring would be reviewed). Scoping reviews provide an overview of the available research evidence on a topic that is not well-defined (30) and when an overview of the literature has not yet been established. This review provides an overview of the peer-reviewed research on *therapeutic mentoring* by clarifying key concepts, intervention characteristics, and outcomes studied in therapeutic mentoring. It also describes the settings in which therapeutic mentoring has been studied, the mentee populations, mentor background and training, and the effectiveness when available. Rather than seek to preemptively define therapeutic mentoring, and then classify the existing literature, this initial scoping review focused on interventions that have been explicitly described themselves as therapeutic mentoring programs in the published literature.

## 2 Methods

### 2.1 Search strategy

Four online databases were searched targeting therapeutic mentoring interventions by community health workers or other paraprofessionals. The databases were MEDLINE, PubMed, PsycINFO, Web of Science in addition to manually retrieving papers from Google Scholar. The search term “therapeutic mento*” was used. Boolean operators between “therapeutic” and “mento*” were not used because the purpose of this review was to describe how self-designated *therapeutic mentoring* has been studied. No date constraints were applied to the search in any of the databases. Scoping review methodology recommends that the study selection phase be iterative (31); after the search was completed, four additional search terms were included: “therapeutic youth mentoring”, “therapeutic group mentoring”, “Campus Corps”, and “Campus Connections”. The first two were added after seeing these terms in a few of the articles that emerged through the first searches. Campus Corps and Campus Connections were added because evaluations of this program emerged through the initial searches. Because these were labeled as therapeutic mentoring programs, the first author made the decision to include any peer-reviewed articles about this program. The review was conducted using the PRISMA-ScR (Preferred Reporting Items for Systematic reviews and Meta-Analyses extension for Scoping Reviews) checklist. The search was completed in August 2024. Because there were no clear hypotheses and the study was designed to be descriptive in nature, it was not pre-registered. Finally, the scoping review was limited to peer-reviewed literature to ensure a baseline level of scientific rigor and quality in the included studies. This approach allows us to synthesize the established research on therapeutic mentoring and to delineate the emerging field in ways that provide a foundation for future, more comprehensive reviews.

### 2.2 Inclusion and exclusion criteria

The purpose of this review was to focus on peer-reviewed articles that explicitly examined therapeutic mentoring for youth mental health. Inclusion criteria for the review were: (1) therapeutic mentoring was noted in the abstract or title, (2) the article was published in a peer-reviewed publication, (3) studies focused on mental health outcome for youth, (4) studies took place in the US, and (5) written in English. Exclusion criteria for the review were publications that: (1) only described therapeutic mentoring practices without a research question, (2) focused on academic outcomes, (3) were unpublished dissertations and theses.

**Figure 1:**
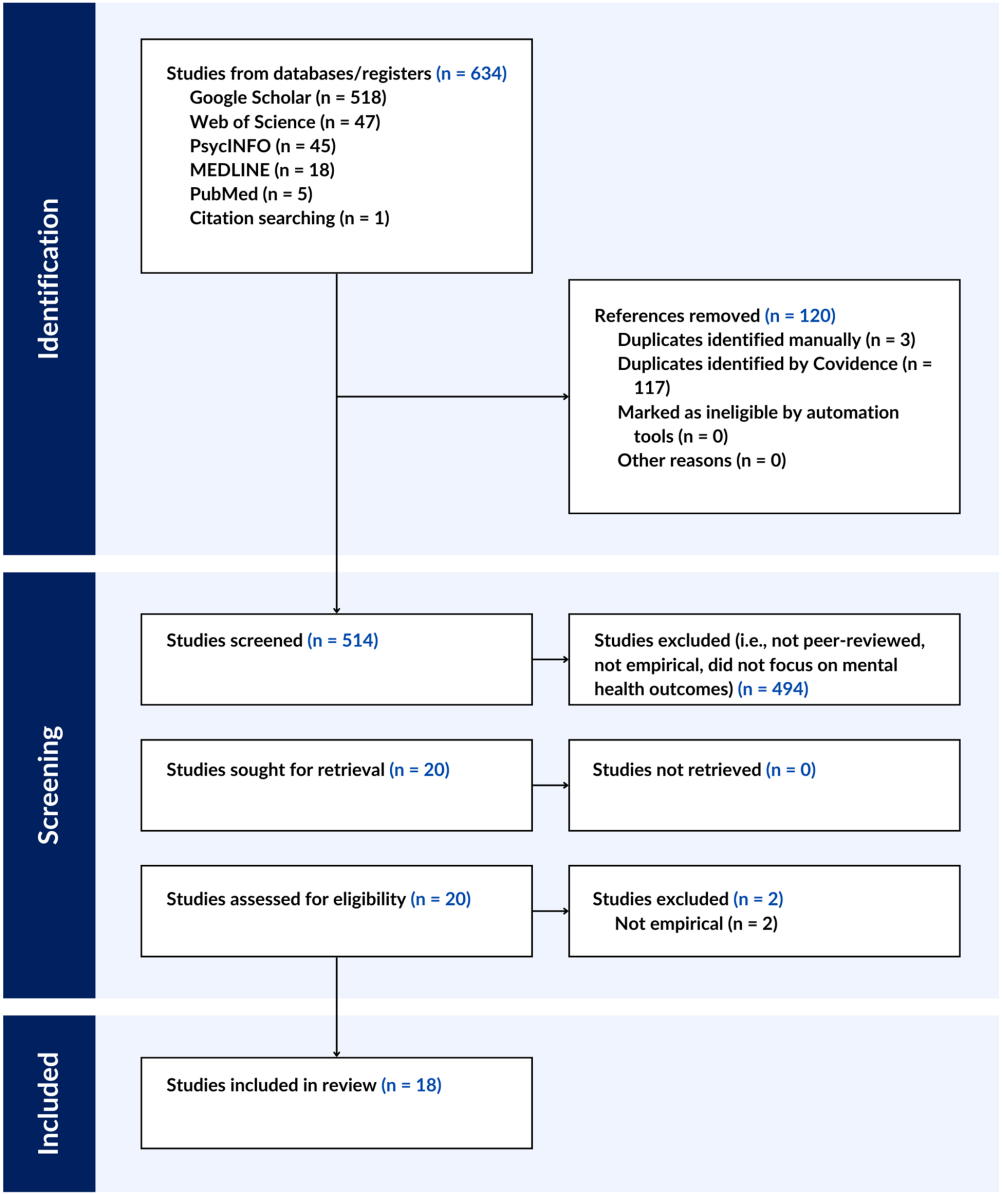
PRISMA flow chart..

During the screening phase, the first author screened for inclusion and exclusion criteria. During the full text review phase, the first author excluded two articles because although they appeared to meet the criteria for inclusion, they were not empirical research articles. See Figure 1 for PRISMA flow chart and Supplementary A for PRISMA-ScR Checklist.

### 2.3 Data extraction

Articles were imported to Covidence for screening, review, and extraction by the authors. Data were extracted by AW and HB into a custom Covidence template that included: article title, year published, aim of study, study design, description of therapeutic mentoring intervention, theoretical orientation of therapeutic mentoring, description of mentor training, description of supervision of mentors, description of mentors, description of mentees, description of study results. To reduce interpretation biases, manuscript excerpts that answered each question were directly copied and pasted in full sections (with citation and minimal syntax edits). After jointly extracting data from the first article, the authors determined that the data were unambiguous so data were independently extracted from the subsequent 17 articles. See Supplementary B for data extraction table.

## 3 Results

A total of 18 peer-reviewed empirical articles examining therapeutic mentoring published from 2003 to 2024 were identified.

### 3.1 Study characteristics

Two studies were randomized controlled trials [RCTs (32, 33)]; two were case studies (34, 35), two were non-randomized experimental studies (36, 37), four were single group pre- and post-survey design (28, 38–40), one was a mixed methods design (41), and seven were qualitative studies (27, 42–47). Of these, thirteen (28, 32, 33, 37, 39, 40, 42–47) examined Campus Connections (CC), a university-based therapeutic mentoring program.

### 3.2 Background of youth served

In all of the studies, youth in therapeutic mentoring programs were from diverse backgrounds and had faced adverse experiences (e.g., Department of Human Services involvement, juvenile justice involvement, behavioral and/or emotional concerns) or came from “at-risk” backgrounds, described below. Most studies reported age ranges that fell within the 10- to 18-years-old range, with the exception of one study that included a range that went to 19-years-old (33).

Youth served in three studies (34, 36, 38) were in foster care, and youth in (38) were described as at-risk of placement disruption. Youth in studies (36, 38) were majority African American and Hispanic; study (34) did not provide demographic data. In a case study with two youth (5), both youth were Hispanic and male. Study (41) described youth as “95% of the mentees were people of color”.

The youth participating in CC came from diverse backgrounds and many had faced adverse experiences, leading to their classification as “at-risk” in the studies. These youth were often referred from various sources within the juvenile justice system, such as the District Attorney's Office, Probation Department, and Department of Human Services (37). School districts also played a role in identifying youth who might benefit from the program, often recommending those struggling academically and considered vulnerable to negative outcomes like school dropout, substance use, and criminal behavior (37). Notably, most youth in the program had at least one formal charge within the juvenile justice system (37). Several studies (28, 32, 33, 37, 39, 41–43, 47) provided some demographic information about the youth served; the majority were male and approximately half were White. Across studies, youth were ages 10- to 18-years-old.

### 3.3 Therapeutic mentoring interventions

Of the three studies focused on youth in foster care, therapeutic mentoring was provided to youth already receiving individual counseling services (36) or youth involved in their local System of Care (SOC) in conjunction with other social services (36–38).

The earliest empirical study identified in this review (34) was a case study of culturally-congruent therapeutic mentoring for young African American males. Therapeutic mentoring was provided in a group setting weekly and was focused on building strong relationships between the mentees and mentors. The mentors served as positive adult role models with whom the boys could experience healthy (as the authors described, “non-abusive and non-exploitative” and consistent) relationships. The intervention itself was centered on African American culture, incorporated African cosmology, and was culturally congruent with the African ethos.

The two studies described therapeutic mentoring as a service provided by the SOC (36, 38). Therapeutic mentoring was defined as a structured intervention featuring carefully vetted mentors who receive continuous supervision and training from master's-level clinicians (38). These mentors were trained and compensated to provide therapeutic support to youth with traumatic experiences and the service was delivered weekly in coordination with other social services, such as family therapy and case management. Termination was always carefully planned. In both studies, therapeutic mentoring was recommended after a clinician assessment (36, 38). Study (36) described the implementation of therapeutic mentoring; mentors and mentees met in person regularly, for 3–5 h each week at a consistent time. Activities were primarily planned by the mentors and mentees, with mentors trained to focus on interactive experiences that emphasized relationship-building. Mentors were instructed to engage youth based on their interests, use open-ended questions, and offer choices to empower mentees.

In the case study of two male, Hispanic teens (35), therapeutic mentoring was referred to them by their therapist. Both youth had single mothers and were receiving services from a clinic employing long-term, home-based psychotherapy program. The therapists' roles extended beyond traditional psychotherapy, involving broader social system consultation. Treatment approaches combined support, system advocacy, and parent empowerment. Therapeutic mentoring involved identifying specific skill deficits in the youth's treatment plan developed collaboratively by mentors and therapists. The process was guided by ongoing consultations between therapeutic mentors and psychotherapists who selected interventions designed to address identified deficits in the youth's functioning. This integrated approach aimed to provide comprehensive support and skill development within the youth's broader social context.

The Arthur Project [TAP (41)]; implemented a therapeutic mentoring program grounded in French et al.'s model (48) for radical healing in communities of color, emphasizing five key components: collectivism, critical consciousness, racial hope, strength and resistance, and cultural authenticity and self-knowledge. The mentoring structure consisted of weekly individual sessions between mentors and mentees, lasting 1–2 h during school hours. Additionally, mentors facilitated small group sessions, referred to as “mentor families”, which met for 2–4 h weekly. The program's community-building efforts were reinforced through Saturday activities, which provided experiential learning opportunities and fostered a sense of collective identity. This comprehensive approach aimed to address the multifaceted needs of youth, particularly those from marginalized communities, by integrating therapeutic support with cultural responsiveness and community engagement.

As described above CC is a structured therapeutic youth mentoring program designed to prevent delinquency, promote academic success, and develop social-emotional skills among at-risk youth. The program operates on university campuses during fall and spring semesters, typically running for 12 weeks with weekly 4 h sessions. Youth are matched one-on-one with undergraduate student mentors who youth select from profiles that include information about the mentor's major, interests, and motivations for mentoring. One study describes that mentees are also receiving counseling from graduate-level counseling students, and mentors receive support from their mentee's counselor (45). CC's structure incorporates several key components designed to foster positive youth development. Each session includes a 30 min walk and talk around the university campus, allowing mentees to explore higher education opportunities while building relationships with their mentors. This is followed by an hour of individualized academic support, where mentors provide tutoring and assist with homework, study skills, and career planning. Participants then share a 30 min family-style meal, fostering a sense of community. The remainder of each session is dedicated to two 45–60 min blocks of prosocial activities, such as sports, art projects, or social justice discussions. One of the studies (33) examined the effect of adding mindfulness to CC programming.

In all but one study (41), youth were engaged with a system of care and referred to therapeutic mentoring (e.g., 34, 36, 38) or were from marginalized backgrounds and simultaneously received counseling through the program (i.e., CC programming). One program (41) was unique in that youth engaging in TAP were not necessarily already receiving services or in counseling/therapy, however the therapeutic mentors were actually social work interns.

### 3.4 Mentor background, training, and supervision

One study did not include information about the therapeutic mentors, including their training (45). These mentors were supervised by therapists providing services to the same youth.

In Utsey's case study (34), mentors were volunteers from a historically Black, college-based social fellowship. They were all African American college graduates with professional jobs and underwent background checks. Mentor training was led by a mental health provider and agency coordinator. Training topics focused on fostering healthy relationships, modeling prosocial behavior, leading discussions on relevant topics (e.g., sexuality, foster care, education), and organizing recreational activities. They also included lessons on fostering a group mentoring experience rather than a traditional one-to-one mentoring model, supporting the children's ability to remain in the community (as opposed to being placed in more restrictive settings), working with youth who had histories of abuse and neglect, and understanding the developmental and emotional issues faced by this population (specifically children in foster care). The training served two main purposes: first, as an educational resource for the mentors, and second, as a means to establish relationships between the mentors and mental health personnel. Mentors were encouraged to reflect on their experiences in African American family and community contexts and to discuss important male kin relationships during training. Ongoing quarterly training addressed youth progress and crisis intervention strategies. Challenges, such as deteriorating youth behavior and complex discussions on personal topics, were discussed during these sessions for further guidance. Mentor supervision was not described.

Therapeutic mentors for foster youth (36, 38) were screened employees with a minimum of a high school education (although some had undergraduate or graduate degrees in social sciences). They completed a 2–3 h orientation with their master-level supervisor, ten hours of additional training within the first six months of their employment, and ongoing trainings while employed. Mentors were trained to take a strengths-based approach and were taught basics of mental healthcare (e.g., encouraging constructive behaviors, professionalism, boundaries, crisis intervention, abuse and neglect reporting (36). They also learned how experiences in foster care could affect youth's emotional expression. Both studies (36, 38) described that mentors were supervised by masters-level clinicians at least once a month either in person or via phone or email. Study 32 also described that mentors kept weekly logs of meetings with mentees that were shared with supervisors.

Mentors from the TAP program were social work clinicians-in-training serving as therapeutic mentors for their clinical internship hours (41). The study described that the majority of the mentors identified with communities of color. Mentors were screened and selected based on their knowledge of social justice and therapeutic mentoring, and received weekly training that included healing-centered engagement, mentoring theory, social justice, and crisis intervention. They were supervised by individuals with LCSW and/or a doctorate in social work and sociology. Mentors submitted regular reports on their mentees' growth.

The CC (27, 28, 32, 33, 37, 39, 40, 42–44, 46, 47) mentors were undergraduate students enrolled at the program's host university (CC study 41 did not describe the mentors). These students were from various academic disciplines, and underwent extensive training prior to engaging in therapeutic mentoring relationships with mentees. The mentors were volunteers who were rigorously screened to ensure compatibility with mentees. The training process for mentors was described as extensive, involving approximately 20 h of training, which was designed to equip mentors with the necessary skills to work with at-risk youth, emphasizing a trauma-informed, strengths-based approach. Mentors received instruction in areas such as active listening, therapeutic communication, boundary-setting, and cultural competency. Mentors were also trained to identify and address risk factors in youth, including those related to mental health, substance use, and family dynamics.

Mentors were paired with supervisors, who were often professionals or graduate students with clinical or mentoring experience, who provided ongoing support and guidance throughout the duration of the mentoring relationship. Supervision included regular meetings where mentors could debrief their experiences, discuss challenges, and receive feedback. These supervisors monitored the mentors' progress, ensuring that they adhered to the goals of the program and maintained appropriate boundaries. In two studies (27, 44), mentors also received peer supervision, where they had the opportunity to collaborate and problem-solve with other mentors in the program.

### 3.5 Outcomes

Only two RCTs were conducted within the identified studies. Both were within CC programs (32, 33). The first (32) examined youth outcomes (e.g., mental health symptoms, socioemotional competencies) after randomly assigning mentees to group-based therapeutic mentoring or one-to-one therapeutic mentoring. Results revealed no significant differences between conditions, however both groups showed improved mental health symptoms. The second study (33) examined the feasibility and efficacy of integrating a mindfulness intervention through CC, comparing CC alone and CC plus a mindfulness-based intervention (MBI). Adding an MBI to CC programming did not have an effect on program attendance and was associated with slightly stronger program acceptability. Effects of adding the MBI on mental health measures were mixed.

In a non-randomized study examining foster youth who received therapeutic mentoring for 18 or more months to those who did not, those youth who had therapeutic mentors had lower stress symptoms than their non-mentored peers (36). Results of a single-group study examining the effect of therapeutic mentoring dosage for foster youth found that youth who received more therapeutic mentoring had lower trauma symptoms and higher family and social functioning, compared to youth with no or fewer interactions with therapeutic mentors (38). In the evaluation of the TAP program (41) that examined pre- and post-program mental health symptoms, the results suggested that youth's depression and PTSD symptoms were higher after participating in the program; authors commented that they thought perhaps youth were more likely to be honest about their symptoms after participating in the program.

In a non-randomized quasi-experiment of CC, youth enrolled in CC were compared to youth who were eligible for CC but were not able to sign up because the program was full (37). Youth who participated in CC reported engaging in problem behaviors less frequently than their peers who did not participate in CC, and also reported lower acceptance of engaging in delinquent and substance abuse. A single-sample study comparing youth participating in CC found to norm-referenced data found that youth participating in CC showed improvement in self-reported conduct problems, negative affect, attention, and social and academic functioning (40).

Qualitative studies of CC found that mentors: experienced personal growth and professional development as a result of participating in CC (27); reported that their relationship with their mentee allowed them to be resilient in the face of transitioning to virtual mentoring during COVID-19 (44); and were able to describe their ethical development (46). Mentees reported that having a mentor through CC allowed them to build resilience and helpful coping strategies (47). Those mentees also reflected that by building a trusting relationship with their mentors, mentees were more likely to be able to trust other adults in their lives. Finally, two studies examined the alliance or relationship quality between mentors and mentees in single-group design studies (28, 39).

## 4 Discussion

This scoping review synthesized the current state of research on 18, empirical, peer-reviewed programs that described themselves as *therapeutic mentoring* interventions for youth.

Several key findings emerged, including the surprising lack of randomized controlled outcome studies on the overall efficacy of therapeutic mentoring as identified by the studies' authors. Based on this review, there are no peer-reviewed studies that have been published that have rigorously tested any model of self-designated *therapeutic mentoring*. The two RCTs included in this review tested within-program characteristics of CC (32, 33), which, while important, do not provide information on the efficacy of therapeutic mentoring as a mental health service for youth. A few of the studies identified in this review showed promising effects (36–38) in non-experimental design studies. However, one single-group study actually found that mental health symptoms worsened after engaging with therapeutic mentoring (37, the Arthur Project). As the authors note, these findings may have emerged as a result of the youth feeling more comfortable disclosing mental health symptoms as they built trust in the program. Particularly given the rising interest in therapeutic mentoring as an intentional practice to reduce the burden of youth mental health challenges, the findings highlight the need for rigorous, carefully designed experimental studies.

Second, we found racially and ethnically diverse youth were over represented in the studies. There are critical gaps in service delivery for racially and ethnically minoritized youth (49). In addition to a global shortage of child mental health providers, there is a particular shortage of providers from diverse backgrounds in particular, limiting the range of perspectives, experiences, and talents represented in the field (50). Research suggests that racial congruence in therapeutic relationships can bring comfort, connection, and ease which may lead to better treatment outcomes (e.g., 51, 52). As such, behavioral health providers, including therapeutic mentors, with minoritized identities likely have a unique understanding of factors that may foster trust, acknowledging that which can impact emotional wellness (e.g., discrimination, acculturation, historical trauma) and promoting better engagement with treatment, and ultimately, better outcomes (53). At least two of the studies identified in this review specifically leveraged therapeutic mentors from similar racial and ethnic backgrounds as the mentees (34, 41). Both of these programs also focused heavily on building mentees' sense of ethnic and racial identity, which is critical to promoting the mental health of diverse youth (54).

Third, therapeutic mentoring programs seem to be theoretically based on mentoring models more so than on psychotherapy models. Although not all studies explicitly stated a theoretical orientation, those that did frequently cited positive youth development frameworks and Rhodes' (55, 56) model of youth mentoring. This model emphasizes the importance of relationship quality in facilitating positive outcomes through enhanced social-emotional wellbeing, cognitive skills, and identity development. Additionally, some programs also incorporated social justice and culturally responsive approaches, particularly when working with marginalized youth populations. Yet, because different interventions are based on different theories and target such dramatically different populations, risks, processes, and outcomes, it is unrealistic to assume that any given conceptual model is sufficiently encompassing and unifying. Rhodes (57) and others have argued more recently that greater theoretical specificity is needed for formal mentoring programs that are designed to achieve specific goals (i.e., mental health). Moving forward, if therapeutic mentoring is seen as a mental health intervention for youth—and consequently, specifically targets youth mental health outcomes like anxiety, depression, stress, and externalizing behaviors—it will be important for therapeutic mentoring models to pull more from evidence-based and culturally-congruent psychotherapy theories and practices.

This is not to say that therapeutic mentors should be providing mental health interventions without the supervision of trained clinicians, however they could easily infuse evidence-based psychotherapy principles into mentoring practices and receive supervision by trained and licensed mental health professionals. In fact, Reach & Rise, a therapeutic mentoring program out of the YMCA, leverages highly-trained mentors to infuse cognitive-behavioral therapy (CBT) practices into mentoring practices. In their technical report, Jarjoura (9) described the evaluation of comparing programs infusing mentoring practices with CBT vs. those who did not on youth outcomes. Results revealed that when mentors were able to implement the CBT practices and when they received support from program staff, youth benefitted. To-date, there are no published peer-reviewed articles describing Reach & Rise, which precluded this program from being included in the systematic review. Likewise, although FHF has undergone extensive RCT evaluation, it does not define itself as a therapeutic mentoring program. In the future it will be important to create a unified definition of therapeutic mentoring that is sufficiently broad that it can incorporate such programs.

### 4.1 Defining therapeutic mentoring

Given the potential of therapeutic mentoring to expand access to mental health services, particularly for marginalized youth populations, a precise definition is crucial for advancing the field of what therapeutic mentoring is and is not. Based on a comprehensive review of the research and core practices of the field, the term therapeutic mentoring describes a distinct type of mentoring program that: (1) explicitly focuses on reducing negative mental health symptoms and increasing psychological well-being and thriving among youth; (2) is delivered by paraprofessionals; (3) uses evidence-based practices to target underlying mechanisms of mental health challenges; and (4) is supervised by highly-trained and licensed mental healthcare providers. If the outcomes of this particular model are clearly defined, the field can efficiently move forward in testing and comparing implementation and delivery of therapeutic mentoring. Critically, the current state of the literature—as evidenced by this scoping review—is fragmented and the programs do not all share a common youth outcome, making it nearly impossible to understand whether therapeutic mentoring “works”.

Requiring that therapeutic mentoring is an adjunct to other established mental health care is less clearcut. In all but one (41) of the studies included in this review, therapeutic mentoring was an adjunct to care youth were either referred by their system of care or case workers, and/or received counseling services in addition to therapeutic mentoring). In (41), social workers in training were the therapeutic mentors; there was no explicit discussion of whether youth received other services during the study period. Although therapeutic mentoring should always be supervised by trained and licensed providers, it is less clear whether youth need to be in other types of mental health supportive therapies to benefit from therapeutic mentoring. In the case of Reach & Rise (9), youth are recruited from the local community; fewer than half of the youth in the evaluation were receiving mental health or behavioral services at the time of participation. Future research needs to evaluate the acceptability and efficacy of therapeutic mentoring as a standalone intervention to reduce negative mental health symptoms and increase well-being.

As described in McQuillin (17), paraprofessional mentors—therapeutic mentors, in this case—can help to scale access to high-quality mental health services. However, because working with youth with mental health challenges will bring risk for programs, it is critical that therapeutic mentors receive training in evidence-based mentoring and basic psychotherapy principles. Moreover, they need to be supervised regularly by licensed clinicians and there needs to be clear rules around documentation.

One of the ways that therapeutic mentoring may be most beneficial would be through the supervised practice of therapy skills in their everyday lives. Supervised practice allows individuals to practice new skills in the company of someone who can monitor their progress and provide supportive, constructive feedback. Although research shows that supervised practice, compared to unsupervised practice, leads to stronger youth mental health intervention outcomes (58), it is difficult for professional therapists to find opportunities to implement supervised practices. Paraprofessionals can fill this void.

### 4.2 Limitations and future research

There are several notable limitations of this scoping review. First, since the purpose of this review was to examine the current state of the peer-reviewed literature on self-designated *therapeutic mentoring* as defined by each studies' authors, there is predictable heterogeneity in program characteristics and the potential for omissions. For example, because Fostering Healthy Futures (29), an evidence-based mental health intervention, does not define itself as a therapeutic mentoring program it was not included in our review. An important next step for this research will be to examine the characteristics and effectiveness of programs that meet the research-informed definition of therapeutic mentoring, iteratively modifying it as new programs and research emerge.

Another potential limitation is that the data extraction was completed by two individuals. However, because both authors copied and pasted text from the articles into the data extraction software, this reduced the potential for authors to bias the results by interpreting and paraphrasing the content. Moreover, both authors were highly trained in research methods (one a clinical psychologist and the other a latter-stage clinical psychology doctoral student).

In addition, because of the heterogeneity of the study designs and outcomes assessed, we are unable to draw firm conclusions about the potential effectiveness of self-designated *therapeutic mentoring* as a practice. Moving forward, for clarity in the field, we argue that only mentoring programs that explicitly target and measure youth mental health and psychological well-being outcomes should be called *therapeutic mentoring*. This will allow for the field to move forward in conducting rigorous evaluations. Research needs to test whether therapeutic mentoring is effective in reducing negative mental health symptoms and increasing psychological well-being when compared to both no intervention and mentoring “as usual” (i.e., mentoring programs that do not explicitly target youth mental health symptoms). Of course, if therapeutic mentoring is found to be superior to no intervention or mentoring as usual, future work should examine how therapeutic mentoring compares to more traditional models of psychotherapy.

## 5 Conclusions

Therapeutic mentoring programs have the potential to be a scalable approach to providing mental health support for diverse youth. The integration of evidence-based and culturally-congruent therapeutic elements within a mentoring framework may also help address some of the limitations of traditional mentoring programs. However, continued research is needed to refine program models, identify key mechanisms of change, and establish best practices for implementation. It will be critical to incorporate feedback from youth and their families as these models are developed to ensure that they are acceptable and culturally congruent, and not simply adaptations of practices that have been evaluated in the context of traditional psychotherapy practice.

Future research on therapeutic mentoring programs should prioritize several key areas. As intervention models are developed, they need to be rigorously examined through randomized controlled trials to establish causal evidence for program efficacy. Researchers should also examine the relative impact of different program components, such as comparing the effectiveness of one-on-one mentoring vs. group activities. Additionally, investigating the optimal duration and intensity of therapeutic mentoring relationships would provide valuable insights for program design. Another important area of focus should be exploring effective methods for training and supporting mentors in delivering therapeutic interventions under the supervision of licensed clinicians. Finally, developing and validating standardized measures for assessing therapeutic mentoring relationship quality and outcomes would greatly enhance the field's ability to evaluate and improve these programs. By addressing these research priorities, scholars and practitioners can work towards developing more effective, evidence-based therapeutic mentoring interventions to support positive youth development and mental health outcomes.

Psychologist John Weisz and his colleagues have proposed a comprehensive strategy for enhancing the efficacy of child mental health interventions, which could serve as a valuable model for the field of therapeutic mentoring (59). This approach emphasizes several key elements: first, identifying and developing programs that effectively address the most prevalent mental health challenges faced by young people; second, ensuring these programs are adaptable and effective across diverse cultural and ethnic backgrounds; third, delineating the specific conditions and contexts in which these interventions are most impactful; fourth, elucidating the underlying mechanisms that drive positive outcomes; and finally, rigorously testing these interventions in various settings before implementing widespread dissemination strategies that ensure accessibility and effectiveness across a broad spectrum of community and clinical environments (59). By adopting this systematic and evidence-based approach, the field of therapeutic mentoring could significantly enhance its ability to deliver effective, culturally sensitive, and widely applicable interventions for youth mental health.
