# Supplementary material for: A scoping review of therapeutic mentoring for youth mental health
Source: Front Child Adolesc Psychiatry. 2025 Jan 27;4:1509971. doi: 10.3389/frcha.2025.1509971 (PMC11841459; doi:10.3389/frcha.2025.1509971)
Supplement: Supplementary file 2 [file Datasheet2.pdf]

Citation	Aim of study	Study design	Describe intervention or therapeutic mentoring program	Describe mentor training	Describe mentor supervision (including credentials of supervisors)	Mentors (description of background + number)	Mentees (description + number)	Describe results of study
Utsey SO, Howard A, Williams III O. Therapeutic group mentoring with African American male adolescents. J Ment Health Couns. 2003;25(2):126-39.	...to demonstrate the clinical utility of this unique and culturally congruent approach to working with a population historically underserved by the mental health profession (i.e., African American male adolescents).	Case study	The therapeutic mentoring group model is based on five principles that guide the interactions between the mentors and the adolescent group members (mentees). They are: Group above self, Respect for self and others, Responsibility for self and community, Reciprocity, Keeping it real (authenticity). The group principles were given to the members at the outset and reinforced at every opportunity. The principles are not only intended to provide a framework for the mentoring relationship, but are tools for behavior change . . . Group therapy sessions were conducted on a weekly basis, and the youth were engaged in discussions around a number of relevant topics (e.g., sexuality, substance use, foster care placements, families of origin) . . . In addition to the specific group activities, the interpersonal relationship between group members and the mentors was important for positive behavior change in the members. Most of the group members had never experienced healthy (i.e., non-abusive and non-exploitative) and consistent relationships with positive Black male figures. For most of the mentees, previous encounters with Black males had been abusive, antagonistic, or adversarial. A number of group members actually voiced their admiration and respect for the mentors and what they stood for in their role as positive Black male role models. For example, one group member remarked it good to see they care bout us, and they doing stuff for kids in the community.	Prior to the beginning of the project the mentors participated in several didactic training sessions conducted by the mental health provider and the agency coordinator. The training covered several pertinent topics germane to implementing the project goals: (a) developing a group mentoring experience for the youth, as opposed to the more traditional one-to-one mentoring concept; (b) helping to maintain the children in the community, as opposed to a more restrictive setting; (c) working with youth who have a history of abuse and neglect; and (d) understanding developmental and emotional issues faced by this population (i.e., children in foster care). The objectives of the training sessions were two fold. First, it was an educational process for the mentors. Second, it allowed the mentors to develop a relationship with the mental health	[Author note: not reported]	Mentors were volunteers from a historically Black, college-based social fellowship.The organization goals include promoting a sense of cultural pride, providing community services, striving for academic excellence, and social responsibility. There were six to eight active mentors involved in the project. All of the men were of African American descent; all were college graduates with professional jobs. In addition to the eight active mentors, other members of the social fellowship were involved at various levels in many of the activities hosted by the agency. All of the mentors underwent criminal background checks including child abuse clearances. The primary role of the mentors was to engage the members in healthy interpersonal relationships, model pro-social behavior, lead discussions regarding issues pertinent to the members (e.g., sexuality, foster care, education, career), and organize bi-monthly recreational activities. The mentors were not responsible for the group therapy component of the mentoring project, though on occasion	Potential participants for the mentoring group project were initially referred by their foster-care caseworkers or by members of the agency mental health division. The mental health service provider interviewed potential members individually to determine their appropriateness for participation in the mentoring group project. The criteria used for the selection of participants to the mentoring group included: (a) male between 12 and 16 years; (b) receiving individual counseling services; (c) if applicable, compliance with medication regime; and (d) expressed support of the foster parent. It should be noted that the group had an rolling admissions policy, and an individual could be referred for participation at anytime . . . [case study] D. D, age 14, and his 13-year-old brother J. D. were original members of the therapeutic mentoring project. They were placed into foster care due to their mother chronic substance use.	Based on anecdotal evidence from the case study presented in the current article, the therapeutic mentoring group was effective in facilitating change in the attitudes and behavior of several group members. More importantly, all of the members were able to experience positive and healthy relationships with Black male role models. The consistency of the mentors, behaviors and presence was a major factor in the group effectiveness.

				personnel. Moreover, the training sessions allowed the mentors to explore the psychological impact of their own personal adversities and to articulate their expectations for the project. The mentors were encouraged to reflect on their own experiences in the African American family and community and discuss relationships with male kin (including fictive kin) who helped shape their own manhood and community integration. Ongoing training sessions were conducted on a quarterly basis. There was frequent communication between the mentors and the facilitator about the youths, progress, crisis situations, and developing strategies to intervene with particular youth in crisis. During several of the training sessions, the mentors discussed their frustrations with several of the youth whose behavior deteriorated while participating in the project. Additionally, the		they were invited to sit-in on a (group) session. The mentors had complete autonomy in the design and implementation of the recreational component of the mentoring project. Recreational activities included, but were not limited to, outings to cultural sites, trips to local parks for sports-related activities (e.g., basketball, football), dining at local restaurants, movies, and in-house activities (e.g., pizza parties, video parties).		

				mentors looked for guidance in some of their discussions with the youth (e. g., the youth were always interested in understanding the mentors, relationships with women).					

Johnson SB, Pryce JM, Martinovich Z. The role of therapeutic mentoring in enhancing outcomes for youth in foster care. Child Welfare. 2011;90(5):51-69.	This study aims to address some of the gaps in the literature ... by examining the effectiveness of TM relationships of varying duration among foster youth. ...to evaluate whether TM is associated with increased youth functioning, groups of foster youth (n=262) were compared based on the presence and extent of TM received between outcomes assessments.	Single group study	In the current study, which took place in partnership with the child welfare system, the length of the matches was dependent primarily on state mandates. However, despite the fact that this could not be controlled for the purpose of the research, it is helpful to observe how the relationship duration impacts outcomes for these foster youth.<br><br>TM, as defined in this study, has several distinguishing characteristics, including carefully screened mentors who receive ongoing supervision and training from master level clinicians (i.e., mentors are trained and compensated to therapeutically respond to youth with traumatic experiences). The mentoring service is provided on a weekly basis in conjunction with other social services, such as family therapy and case management, and the termination process is carefully planned and implemented.<br><br>The intervention took place within a social service agency in a large metropolitan area in the Midwestern United States under the System of Care (SOC) program, a service contract between private agencies and the state Department of Children and Family Services (DCFS). The SOC program provides in-home family and individual counseling, TM, advocacy, case management, and referral and linkage to foster families referred by their DCFS caseworkers for being at risk of placement disruption. The SOC service is a short-term, crisis intervention service. Services were provided on average for six to nine months.<br><br>TM was available to families identified by an SOC clinician as potentially benefitting from	They received an individual orientation (two to three hours) with their assigned supervisor (a master level clinician), and participated in at least 10 hours of training within their first six months of employment, as well as ongoing training throughout their tenure at the agency.	Mentors had contact with their supervisor and the mentee clinician a minimum of once per month by phone, by e-mail, or in-person to discuss mentoring relationship progress and any problems or issues that arose with youth.	Therapeutic mentors were screened extensively prior to hiring and had a minimum of a high school education, and often some undergraduate or graduate study in the social sciences.	The sample consisted of 262 youth living in foster care who were assessed as being at-risk of placement disruption by their DCFS caseworker. A majority of the youth who participated in the current study were African American (76%), followed by Hispanic (9.9%), Caucasian (5.3%), multiracial (4.2%), other (3.1%) and unknown (1.4%). A majority of the youth were between 6 and 15 years old.	Evidence from the analyses revealed that, particular to the irst six months in the program, (1) foster youth who received a substantial amount of TM improved significantly on measures of family and social functioning, school behavior, and school achievement relative to foster youth who received a limited amount of TM; and (2) foster youth who received limited TM showed a lack of improvement overall on measures of family and social functioning, school behavior, and school achievement relative to youth who received either substantial TM and no TM. Further, for those foster youth who received substantial TM between 12 and 18 months in the program, a significant decrease in the expression of trauma symptoms was demonstrated as compared to youth with no TM, a group of which worsened significantly after 18 months in these same areas. Significant differences were not found for group comparisons in the other time intervals for the remaining domains examined.

			the mentoring service.<br><br>Once a mentor was identified for each youth, a pre-placement meeting was facilitated in the client home by the clinician to introduce the mentor to the family, review program guidelines, and discuss goals for the mentoring relationship. he mentor and mentee met on a consistent basis (e.g., same day and time each week) for an average of three to five hours each time. Shared activities were primarily initiated and planned within the mentor-mentee relationship; however, mentors were also given the opportunity to seek the support of the clinician and supervisor in planning activities.					

Haddock S, Weiler L, Krafchick J, Zimmerman TS, McLure M, Rudisill S. Campus Corps Therapeutic Mentoring: Making a Difference for Mentors. Journal of Higher Education Outreach and Engagement. 2013;17(4):225-56.	Our study aims to address these gaps in the research literature and to illustrate the outcomes that college student mentors experience when mentoring at-risk youth in the context of a service-learning course.	Qualitative research	A new Campus Corps session begins at the start of each fall and spring semester. The program takes place on the Colorado State University campus, from 3 p.m. to 9 p.m., four days per week. Each youth participant is paired with a college student mentor and each pair attends one session per week; 30 mentor-mentee pairs are present for each day session. Youth select their mentor from Mentor Profiles, which are prepared by the mentors for this purpose. These one-page profiles summarize each mentor interests (sports, music, fun, hobbies, etc.), academic major, personal and professional goals, and why the student chose to become a Campus Corps mentor. To provide additional social support to mentors and mentees, mentor-mentee pairs are organized into Mentor Families, which are comprised of four mentor-mentee pairs organized by youth age and supervised by a Mentor Coach, who is an experienced student mentor. Mentor Families engage in many of the evening activities together. An evening of Campus Corps begins and ends with an hour that only the college students attend. During this time, the family therapist instructor aids the students with the development of plans to best support mentees. This time is also used for group reflection and debriefing of the students, experiences. This level of support is intended to help mentors experience high self-efficacy in their ability to maintain a successful relationship; an integral component of maintaining high-quality mentorship. Youth attend Campus Corps from 4 p.m. to 8 p.m. During this time, mentors and mentees (a) take a walk on campus to re-connect, and learn about campus and various professions, (b) work	Students participate in an extensive 20-hour training program prior to mentoring. The training is conducted by faculty from Colorado State University, juvenile probation officers, investigators from the District Attorney Office, the juvenile court magistrate, and other key juvenile justice professionals. Training includes juvenile court observations, mentoring skills instruction, role playing, instruction in adolescent development, and an orientation to the systems from which youth are referred to the program (juvenile justice, schools, community agencies, etc.). Some students also elect to participate in an optional, intensive training in Motivational Interviewing, which is offered prior to start of each the semester. During the 12 weekly sessions, students receive ongoing training and supervision to continue to support their development as mentors and their acquisition of professional skills.	Therapeutic mentoring supervision is provided by family therapists who are trained in Marriage and Family Therapy. A highly selective group of experienced mentors (Mentor Coaches) provide direct supervision to mentors in a Mentor Family comprised of only mentors. This aims to assist mentors with youth interventions and role modeling. Mentor Coaches are also available to strategize how to best meet the needs of individual youth.	Campus Corps enrolls undergraduate and graduate students from any major on campus. Students from over 40 majors have served as mentors to date. Students must follow a specific process in order to be deemed eligible as a mentor, beginning with attendance at a mandatory informational meeting and subsequent formal application to become a mentor. Applications allow program staff to identify mentors who possess important skills and attributes for effective mentoring, including experience in helping roles, sense of self-efficacy, and appreciation of cultural and socioeconomic differences. If accepted into Campus Corps, students must also complete and pass a criminal background check. Because Campus Corps is offered as a one-semester service-learning course, students receive course credit for participating as mentors in the program. Many students elect to participate in subsequent semesters of Campus Corps, either as a Mentor Coach or as a Research or Teaching Assistant.	Campus Corps serves youth between the ages of 10 and 18. The youth who attend Campus Corps are considered at risk of offending or re-offending and are referred to Campus Corps from a variety of sources including the District Attorney Office, the Probation Department, the Department of Human Services, the local school district, and other community agencies. Seventy percent of youth enrolled in the program possess at least one charge with the juvenile justice system. Although all youth attend voluntarily, Campus Corps is often part of each youth diversion or probationary conditions, or treatment plan.	Specifically, the experience of mentoring at-risk youth within a service-learning course appears to benefit the college student mentors who participated in Campus Corps. Results from this qualitative study revealed that participation in Campus Corps resulted in mentors experiencing: (1) significant personal growth; (2) notable professional development; and (3) valuable civic attitudes and engagement.

			on homework, (c) share a meal, and (d) engage in pro-social activities, such as cooking, sports, and art. Because Campus Corps serves at-risk youth, Campus Corps has created a structure that goes beyond simply spending time together. Campus Corps activities integrate what is being learned (e.g., rules of kickball and how to form clay models) with developmental assets (e.g., teamwork and creativity).<br>Campus Corps is held each semester, with youth attending 12 of the 16 weeks. The first four weeks of the semester are devoted to mentor training.					

Johnson SB, Pryce JM. Therapeutic mentoring: reducing the impact of trauma for foster youth. Child Welfare. 2013;92(3):9-25.	The current study seeks to examine whether youth in foster care who demonstrate significant symptoms of trauma benefit from therapeutic mentoring (TM) as compared to youth who do not receive TM as a part of a larger service delivery system.	Non-randomised experimental study	The SOC service is short-term (lasting on average 6-9 months) and designed to provide crisis intervention through a wide array of flexible services, often in community settings, the foster home, or at school. The two primary goals of the SOC program are to maintain placement stability for foster youth and to improve the youths, emotional and behavioral functioning before discharge from the program. Within the SOC program, therapeutic mentoring was one service available to families. Clinicians referred some of their clients to mentoring after assessing whether the youth had the potential to benefit from a mentoring relationship. This decision was typically made via an initial clinical assessment by the clinician, which included interviews with the youth, foster parents, and caseworker.<br><br>Once the match was made, the mentor and mentee had in-person meetings on a consistent basis (e.g., same day and time each week) for an average of three to five hours each time. Shared activities were primarily initiated and planned within the mentor-mentee relationship; however, mentors were trained to consider interactive activities (e.g., games, cultural events, outdoor activities) that would allow for the emphasis to be on the relationship, rather than on the activity itself. Mentors were also trained on how to engage youth in activities based on the youths' own interests. Guidelines were offered for engaging conversation through open-ended questions, and ways to provide youth with choice of activity in order to empower them to express their own views and desires. The mentors were instructed on how to best respond to both	Therapeutic mentors were extensively screened prior to hiring, and had a minimum of a high school education, and often some undergraduate or graduate study in the social sciences. They received an individual orientation (2-3 hours) with their assigned supervisor (a licensed clinical social worker), and participated in at least ten hours of training within their first six months of employment, as well as ongoing training throughout their tenure at the agency. Training topics included strengths-based approaches to working with children with emotional and behavior disorders, engaging youth in constructive activities, professionalism and boundaries, therapeutic crisis intervention techniques, and abuse and neglect reporting protocol.Training also included specific attention to the abuse and neglect experiences of foster youth and how these experiences may result in	Each mentor-mentee match was monitored and supervised by a Master-level clinician. Mentors had contact with their supervisor and the mentee clinician a minimum of once per month to discuss mentoring relationship progress and any problems or issues that arose with youth.In addition,weekly logs that detailed each mentoring session and its interventions were submitted to the program supervisor, who would then address any issues requiring follow-up with the mentor in a timely manner. Through these supportive contacts, program supervisors also modeled for the mentors empathic and empowering responses, as well as facilitated problem-solving regarding potential abuse/neglect reporting or other conflicts (e.g., sibling issues, negative parent responses to youth behavior, police involvement with family).	Therapeutic mentors were extensively screened prior to hiring, and had a minimum of a high school education, and often some undergraduate or graduate study in the social sciences	The sample consisted of 262 youth living in foster care who were referred to the System of Care (SOC) program at a private agency located in a large urban setting.The SOC program assigned Master-level clinicians to assess and design a treatment plan for foster families referred by their Department of Children and Family Services case-workers for being at risk of placement disruption. SOC clinicians had the ability to offer in-home family and individual counseling, therapeutic mentoring, advocacy, case management, and referral and linkage to community services, depending on the needs of each particular family.Of the 262 youth included in thisstudy who received SOC services, 106 received TM as a part of the service array, and 156 received other services(e.g., case management, family therapy, etc.), but not TM. A majority of the youth who were included in the current study (i.e., mentored and non-mentored youth) were between 6 and 15 years old and African American (76%),followed by Hispanic (9.9%),Caucasian (5.3%), multiracial (4.2%), other (3.1%) and unknown (1.4%).	Analysis suggests that mentored youth who remain in the program longer (up to 18 months) and receive TM improved significantly over non-mentored youth in terms of demonstrating a reduction in the expression of stress symptoms associated with trauma. Specifically, analysis revealed three important findings related to the impact of TM on trauma expression in foster youth. First, the results from the Traumatic Stress Symptoms Scale revealed significant differences,suggesting that those youth receiving substantial TM improved significantly more than those not receiving TM in this area. Second, the group of youth who did not receive TM worsened from baseline to 18 months on the Traumatic Stress Symptoms scale, while also experiencing an increase of trauma experiences during the same period.Third, after 18 months in the program, those youth who did not receive TM showed declines, as evidenced by the increase in the expression of trauma, such as re-experiencing (i.e., intrusive memories of a traumatic event) and dissociation.

			appropriate and inappropriate requests by mentees, including behavior management and therapeutic crisis intervention training. Finally, mentors were required to give youth several weeks' notice when it was time for the mentoring relationship to end, and to be sensitive and empathic about the loss this would likely present for the youth, who often became very connected to their mentors.	expression of trauma symptoms. Mentors were instructed on how to respond sensitively and therapeutically to these symptoms in order to help youth express themselves safely and productively.				

Desmarais R, Sacco-Dion C, Sacco FC, Decoteau N. Delinquency Diversion using Home-based Psychotherapy and Therapeutic Mentoring. International Journal of Applied Psychoanalytic Studies. 2014;11(2):163-71.	The cases described in this paper illustrate how long-term psychotherapy and a strong mother can combine to divert a young child who displayed behavioral problems in first grade.	Case study	The two cases involve single mothers of color and one clinic with multiple therapists and a unifying philosophy of long-term, home-based psychotherapy. The role of the therapist can be seen to involve more than just pure psychotherapy. The therapist uses consultation to create involvement with the family entire social system from court, state agency, and most importantly through school. The parent used the psychology department to advocate for special education support for disruptive behaviors. The treatment courses for each youth offered a blend of support, system advocacy, and parent empowerment.<br><br>When the therapist makes a referral for a therapeutic mentor, the specific skills deficits are identified as part of a treatment plan that is developed by the mentor and informed by the therapist. This pattern of involvement begins with a thorough clinical assessment shared by the clinician who then identifies with the mentor help areas that can be buttressed using the tactics and techniques of therapeutic mentoring.<br><br>TM targets skill building in these areas and follows a distinct plan guided by the consultations between the therapeutic mentor and the psychotherapist. Together, this team generates information, activities, parental suggestions, school interventions, and community activities that address the deficits that we will outline.<br><br>[Author note: This manuscript includes additional details of the therapeutic mentoring intervention, however have been omitted from this table owing to space constraints.]	[Author note: Not included]	Therapeutic mentor supervisors [not described]... consultation with therapists	[Author note: Not described]	Seventeen year-old Latino male first identified with emotional and behavioral problems upon entering school. He is the eldest and only child to his parents who parted ways when he was about age five. He has no contact with his father. He currently resides at home with his mother, a single parent, and his 10 year-old twin half-siblings whose father remains involved on some weekends. The majority of his education has been provided in a public therapeutic day school setting as part of a special education plan.<br><br>Twelve year-old Hispanic male first identified with emotional and behavioral problems upon entering school at age five. He is the only child of his parents. He resides in a single family home with both of his parents and his grandparents. He was transitioned to a therapeutic day school designed to provide public education for those students who cannot be safely maintained within the traditional public school setting.	The youth will graduate and not experience any out-of-home placement, hospital admissions, or incarcerations. The families remained intact and functioning in the community despite early indicators of risk. The mothers acted early and stuck with it with the support of the clinic therapists, medical and psychological back-up, and a responsive administrative staff supporting the professional staff and clients.

Weiler LM, Zimmerman TS, Haddock S, Krafchick J. Understanding the Experience of Mentor Families in Therapeutic Youth Mentoring. Journal of Community Psychology. 2013;42(1):80-98.	In sum, the primary benefit of Mentor Families is likely to be the support and opportunities provided to each dyad by way of the mentor, intentional mentoring community, and supportive hierarchy structure of Campus Corps. Through an examination of mentors, and mentees, experience of Mentor Families, we shed light onto one of the many social ecologies involved in mentoring practice... Results from this preliminary, grounded theory study enable us to determine the potential of this novel mentoring component and whether it warrants further investigation.	Qualitative research	Campus Corps: Therapeutic Mentoring of At-Risk Youth is a youth mentoring program at Colorado State University, informed by Rhodes, model of mentoring (2002, 2005). Campus Corps provides one-to-one mentoring within Mentor Families and a supportive mentoring community to youth at risk of entering the juvenile justice system and first-time offenders, aged 10-18 years. Youth are referred from community agencies involved with the juvenile justice system. Families of the youth are involved and supported. Prior to enrollment, all youth and their families participate in an extensive intake interview with a Campus Corps intake worker to review program requirements and connect the family to other community services, as needed. The program aims to prevent delinquency and recidivism, while addressing relevant correlates (e.g., substance use) and promoting important protective factors (e.g., academic success). Campus Corps was designed in direct response to a call to action from the local juvenile justice system . . . Campus Corps utilizes college student mentors to directly serve approximately 280 at-risk youth on campus each year . . . Campus Corps operates for 12 weeks during the fall and spring semesters for four sessions per week (Monday-Thursday) from 4:00 to 8:00 p.m. Approximately 30 youth and 30 student mentors, eight mentor coaches (experienced undergraduate student mentors), and one family therapist supervisor participate each night. Each evening, Mentor Families participants first participate in intentional walks around the university campus. During this walk, youth learn about opportunities within higher	At the center of the program is a three-credit service-learning course in which college students are selected through a competitive application process, screened for work with at-risk populations, thoroughly trained, and closely supervised to maximize the effectiveness of their mentoring. The service-learning course combines 20 hours of training prior to mentoring and weekly mentoring sessions with academic readings and reflective assignments. Additionally, for one hour before and after the weekly meeting, students think critically and discuss relevant issues for at-risk youth and their families, analyze and apply models of adolescent development, strength-based approaches, and social justice to their own life experiences as well as their mentees, life, and discuss and apply theories of best practice mentoring. Finally, in line with service-learning best practices, students are assessed	Mentor Families provides built-in programmatic support through the support of fellow mentors and an experienced mentor directly supervising the Mentor Family . . . one family therapist supervisor participate each night . . . In this situation, faculty and graduate students may serve as the clinical facilitators and supervisors...	All adult participants were enrolled as mentors in the Campus Corps program during fall 2010 or spring 2011. Mentors entered the research study on a voluntary basis after obtaining consent for their participation. In all, 212 mentors of the possible 225 agreed to participate in the study, ranging in age from 18 to 50 (M = 21.30, SD = 4.26). Thirteen percent of the participants were male and 87% were female. The majority of the sample primarily identified as Caucasian (89.5%), with fewer Latino (3.8%), American Indian or Alaskan Native (2.6%), African American (2.2%), and Asian (1.9%) participants. Additionally, most participants (51.9%) were seniors, 45.6% were juniors, and 2.5% were sophomores. Finally, as can be expected given the nature of the program, a majority of the participants (42%) majored in psychology, with 29% majoring in human development and family studies, 14% in health and exercise science, and less than 5% in Spanish, family and consumer sciences, and other majors.	All youth participants were mentees enrolled in the Campus Corps program during fall 2010 or spring 2011. All participants of the Campus Corps program during these two sessions (111 youth from the fall program and 114 youth from the spring program) were recruited for the present study. In all, 87 youth participants entered the research study on a voluntary basis, upon obtaining parental consent and youth assent. Because of a greater proportion of male youth in the overall Campus Corps program, there were more male participants (64%) in this research study than females. Youth participants ranged in age from 10 to 18 years (mean [M] = 15.21, standard deviation [SD] = 1.93). The race and ethnicity of the participants was similar to the racial demographics of the area, with the majority of the sample primarily identified as Caucasian (52%), with fewer Latino (37%), American Indian or Alaskan Native (1.4%), African American (1.2%), and other (3.8%) participants. Some participants chose not to identify a race or ethnicity (4.6%). The demographic composition of the youth who volunteered to participate in the present study was similar to that of the	95% of the mentoring relationships were sustained through the program and it appears that Mentor Families may play a role in aiding in this success. Specifically, mentors and mentees report that Mentor Families provides a place in which they can (a) receive support and supervision, (b) belong, and (c) grow and learn . . . The themes revealed in the current study point to the importance of a place. Specifically, Campus Corps and Mentor Families occupy not just a physical space (e.g., campus, classrooms, family tables) but also an emotional space (e.g., warm, trusting relationships). Youth mentoring is often a solitary activity that occurs in isolation, away from other mentor-mentee pairs. Mentor Families and a structured mentoring community may provide additional support for the pair by having a consistent physical and emotional space youth and mentors can come to week after week.

			education and various majors and careers while getting some exercise. This time is also important for mentees to catch up with their mentors about their week while building the mentoring relationship. Next, mentors provide individualized tutoring for their mentee. Mentors help their mentees with school homework, study skills, goal setting, and career planning. Youth grades and attendance are monitored each week and communicated to family and referral sources in a weekly progress report. A nutritious dinner is provided by the local food bank and is enjoyed every night together within the Mentor Family. The remainder of the evening is broken into two one-hour blocks where mentor and mentee dyads engage with other dyads in a variety of prosocial activities (e.g., art projects, sports, writing, dance, and science experiments) designed to provide an alternative to delinquency. These activities support the development of new interests, hobbies, or social competencies and help youth gain confidence in a variety of skills. Throughout the evening, mentors serve as role models while empowering youth through fostering a sense of agency and purpose in their own lives.	through weekly reflection journals and group reflection activities, professional case documentation, activity planning and implementation, writing assignments, and direct observation of mentoring practices			overall Campus Corps program population.	

Weiler LM, Haddock SA, Zimmerman TS, Henry KL, Krafchick JL, Youngblade LM. Time-limited, structured youth mentoring and adolescent problem behaviors. Appl Dev Sci. 2015;19(4):196-205.	... the following description of Campus Corps is included to introduce the theoretical underpinnings of the program and to provide a context for our hypothesis: Controlling for pre-intervention differences, baseline scores, and key covariates, youth who participate in Campus Corps will report lower frequency and acceptance of problem behavior and higher peer refusal skills and level of autonomy from substance use at posttest than participants in the comparison group.	Non-randomised experimental study	Campus Corps is a structured, time-limited (12-week) mentoring program for high-risk youth (ages 11-18), including those at risk for entering the juvenile justice system and youth formally charged with an early offense. Campus Corps provides prevention and early intervention services prior to deeper involvement in juvenile justice, school dropout, or serious behavioral health problems. Campus Corps, mentors are undergraduate university students enrolled in a 3-credit service-learning course which includes mentor training, live supervision, and ongoing support	Campus Corps, mentors are undergraduate university students enrolled in a 3-credit service-learning course which includes mentor training, live supervision, and ongoing support . . . oundational to Campus Corps is the intentional multi-level mentoring community. Each mentor-mentee relationship is a part of a Mentor Family (i.e., small groups of 4 mentor-mentee pairs) nested within the larger mentoring community which is further supervised and supported by Mentor Coaches (i.e., experienced youth mentors) and family therapist instructors (i.e., graduate students trained in systemic thinking and therapeutic interventions who oversee the mentoring community and service-learning course). Added benefits may occur as a result of combining one-on-one mentoring within a structured group setting. Through the primary mentor-mentee relationship, as well as additional	Additionally, the intentional structure of Campus Corps is designed to provide in-the-moment support and live supervision for mentors. Mentors rely on fellow mentors, their Mentor Coach, or the instructor to alleviate pressure to be all-knowing, to support behavioral interventions, and to model positive relationships . . . Campus Corps, mentors are undergraduate university students enrolled in a 3-credit service-learning course which includes mentor training, live supervision, and ongoing support.	Campus Corps, mentors are undergraduate university students enrolled in a 3-credit service-learning course which includes mentor training, live supervision, and ongoing support	Campus Corps participants (n=187, 63.1% male) . . . comparison condition (n=128, 66.4% male) . . . Participants were recruited to participate in this pilot study if they were 11 to 18 years old and either had a prior legal offense or were deemed at risk of offending by their agency contact (i.e., professional at the Office of the District Attorney, Probation Department, restorative justice and diversion programs through the Department of Human Services and two local agencies). Community partners were instructed to refer youth at risk for entering the juvenile justice system or youth formally charged with an early offense. Youth were considered at risk of offending if one of six risk factors from the Arizona Needs/Risk Assessment (described below) was present in his or her life. Given the high risk nature of the adolescents served by these community partners, it was not surprising that all youth referred to the study met the inclusion criteria of a prior offense or presence of at least one risk factor. Also not surprisingly, youth at the deeper end of the juvenile justice system (who may not benefit from such a preventive intervention) were not referred by the selected agencies. As noted	Consistent with prior research, mentoring may have a positive, yet modest, impact on the attitudes, behaviors, and identities of youth related to problem behavior. On average, Campus Corps participants reported lower acceptance of problem behaviors, such that they were less likely than the comparison group to perceive delinquent and substance use behavior as acceptable. Campus Corps participants also reported lower frequencies of problem behaviors than youth in the comparison group. No observable differences, however, were found between groups regarding peer refusal skills after accounting for pre-intervention differences. In terms of sense of identity, mentored youth demonstrated a greater sense of autonomy from marijuana use than non-mentored youth, but no group differences were noted on autonomy from alcohol use.

				prosocial peer and adult relationships in the Mentor Family and larger community, youth build positive prosocial relationships and gain a sense of mattering.			below, however, some youth were too young to participate in the study and were excluded from the final sample. Youth were not excluded due to other demographic characteristics, mental health status, or prior offense . . . Of the 382 youth enrolled in Campus Corps during the aforementioned semesters, 74.7% (n = 286) participated in the research study. The remaining youth did not participate due to being 10 years old at the time of the pretest, lack of parental consent, and/or choosing not to participate in the research study. Forty-eight percent of youth recruited to participate in the comparison group consented to research (n = 136). In all, 422 youth consented to participate.	

Haddock SA, Zimmerman TS, Gile Thomas A, Weiler LM, Krafchick J, Fredrickson GJ. A Qualitative Analysis of Mentee Experiences in a Campus-Based Mentoring Program. Journal of Youth Development. 2017;12(4):61-80.	The purpose of this study was to add the voices of mentees to the literature. The mentees in this study participated in a campus-based mentoring program. The mentees were referred through the juvenile justice system, following their first arrest for a low-level crime. The findings reported in this study can inform mentoring programs across the country by providing mentees, own perspectives on how participating in a mentoring program has influenced their lives.	Qualitative research	Entry requirements included that youth were at risk for future delinquency, between the ages of 10 and 18 years, and residing in Larimer County, Colorado. Participants were part of a probationary diversion program intended to reduce the depth of a youth entry into the juvenile justice system by providing opportunities for expunging charges and avoiding adjudication (Chapin & Griffin, 2005). A diversion plan could include several components such as community service, drug or alcohol education, and enrichment activities. One option for a diversion plan is for the youth to participate in the Campus Connections Mentoring Program.<br><br>Campus Connection pairs college student mentors with youth mentees, most of whom are involved with the juvenile justice system. Mentors and mentees meet one day a week, for 4 hours, for 12 weeks on the University campus. Mentor-mentee pairs participate in a community of approximately 25 other pairs and engage in meaningful activities together, including exploring campus during 30 minute weekly walks, called Walk and Talk, getting homework help for an hour each week, eating dinner together, and participating in 2 hours of prosocial activities (e.g. sports, art, cooking).	[Author note: Not described]	[Author note: Not described]	[Author note: Not described]	Out of 225 youth, 87 volunteered to be interviewed. Sixty-four percent of the participants were male, and 36% were female. Participants ranged in age from 10 to 18 years (M=15). The race and ethnicity of the participants was similar to the racial demographics of the geographic area and Campus Connections, with the majority of the sample primarily identifying as Caucasian (52%), with fewer Hispanic (37%), American Indian or Alaskan Native (1.4%), African American (1.2%) and other (3.8%) participants. Some participants chose not to identify a race or ethnicity (4.6%).	Interview responses revealed that youth perceived the program influenced them the most in five key areas: (a) improved academic experience, (b) enhanced relationships, (c) feelings better about themselves, (d) improved future orientation, and (e) decreased likelihood of further delinquency.

Boat AA, Weiler LM, Bailey M, Haddock S, Henry K. Mentor's Self-Efficacy Trajectories During a Mentoring Program for At-Risk Adolescents. The Journal of Primary Prevention. 2019;40(6):575-89.	Our study aims to (a) describe trajectories of mentor self-efficacy; (b) examine how changes in mentor self-efficacy are associated with mentor alliance, a component of mentoring relationship quality, at the end of the mentoring relationship; and (c) examine how mentor personality traits and mentee risk are related to changes in mentor self-efficacy.	Single group study	CC is a 12-week preventive intervention for at-risk youth. Youth selected their mentors from several mentor profiles (i.e., information on the mentor major, interests, and reasons for becoming a mentor) to ensure matches were based on shared interests. An adequate number of mentors were recruited to ensure that all youth were matched one-to-one. Matches spent four hours per week together at the CC facility, along with other mentoring dyads, engaging in a 30-min walk on campus, an hour of academic support, a 30-min provided dinner, and two 1-h prosocial activities (e.g., gardening).	Mentors were enrolled in a service-learning course and participated in 18 h of training prior to the program. Faculty and youth development professionals provided mentoring skill instruction, social justice training, adolescent development instruction, and orientation to systems from which youth were referred.	Throughout the program, faculty provided mentors with ongoing in-person supervision and support including time for preparation and reflection before and after the sessions.	Program staff accepted mentors into the program following an application process and criminal background check. All mentors (N=238) were undergraduate students with a mean age of 20.6 years (SD=2.2). Mentors were mostly female (70.3%). Most self-identified as White (77.3%), 11.4% as Hispanic/Latino, 3.3% as Asian American, 2.9% as African American/Black, 1.1% as other race/ethnicity, 0.7% as Native American/American Indian, and 0.7% as Native Hawaiian/Pacific Islander.	Our study sample included adolescents and mentors from a Western city in the United States. Program staff recruited adolescents for a study of a mentoring intervention known as Campus Connections (CC). Youth eligible for participation as mentees were 11-18 years old, had experienced at least one risk factor as indicated on a baseline risk assessment, and were available to participate in the CC program. Of the 272 mentees recruited, 27 never started the intervention and were not assigned a mentor, and seven mentees did not consent to participate. Of the 238 mentees in the study sample, 61.8% were male, and the mean age of the entire sample was 14.1 years (SD=1.76). A little over half of youth identified as White (55.9%), 27.6% as Hispanic/Latino, 10.7% as multiracial, 0.4% as American Indian/Alaska Native, 4.0% as African American/Black, 0.4% as Asian American, and 1.1% did not report their race/ethnicity. More than half of caregivers reported a household income of 40,000 or below (67.2%).	This study identified three groups of mentors: (1) mentors moderately high in self-efficacy and then decreasingly over time, the decreasing group; (2) mentors relatively high and stable in self-efficacy, the stable group; and (3) mentors high in self-efficacy and even more increasingly so, the increasing group. Mentor self-efficacy should not be assumed to be stable over time and initial self-efficacy is not necessarily reflective of later self-efficacy. While the self-efficacy of most mentors continued at high levels and/or increased even further, a downward trajectory emerged for a group of mentors who reported lower perceived self-efficacy over time. As expected, the three subgroups differed on youth- and mentor-reported mentor alliance. Mentors and youth reported a greater mentor alliance in the increasing relative to the decreasing group. This suggests that growth in self-efficacy is associated with more positive relationships at the end of a mentoring program. Because of the amendable nature of self-efficacy, it may be an important factor for mentoring programs to target as a means for fostering quality mentor alliance. In our study, most mentors retained relatively high levels of self-efficacy throughout a mentoring program designed for at-risk youth. However, some mentors experienced downward shifts. These mentors were more likely to rate themselves as

								experiencing less extraversion, less agreeableness, and less conscientiousness prior to the start of the mentoring program. These mentors may also rate their mentoring relationship less positively at the end of the program.

Weiler LM, Boat AA, Haddock SA. Youth Risk and Mentoring Relationship Quality: The Moderating Effect of Program Experiences. American journal of community psychology. 2019;63(1-2):73-87.	Therefore, this study sought to identify whether, and which, youth mentoring program experiences moderated the negative association between youth level of risk and the quality of the mentoring relationship.	Single group study	Campus Connections (CC) is a mentoring program for youth at-risk for poor developmental outcomes, such as behavior and emotional problems. It is flexibly designed to respond to the needs of a heterogeneous group of adolescents with various risk levels. The program is grounded in theoretical and empirical research on positive youth development settings (Eccles & Gootman, 2002; Kelley, Ryan, Altman, & Stelzner, 2000; Tseng & Seidman, 2007) and Rhodes, model of youth mentoring (2005) . . . CC provides opportunity for each of these components and distributes resources accordingly (e.g., mentor supervision and support, organization of matches, and physical space designated for certain activities). Together, these perspectives support what Cavell and Elledge (2014) later referred to as mentoring-as-context, in which mentoring is used as a context for prevention-focused programming, even in short-term relationships. The program occurred over 12 weeks at a university campus, where youth were matched with an undergraduate mentor. To ensure strong mentor-mentee matches, youth could select their mentors from several mentor profiles, which included information about the mentor major, interests, and reasons for wanting to be a mentor. Mentor-mentee dyads spent one evening (4 hours) per week together. During this time, mentor-mentee dyads engaged in a variety of activities including a 30-minute walk around campus, 1 hour of individualized academic and/or career support, dinner, and multiple prosocial activities (e.g., sports, art, music). Mentees selected activities from a range of options that	Mentors were provided training and supervision opportunities. All mentors participated in 18 hours of training conducted by Marriage and Family Therapy faculty prior to participating in the CC program. Training included juvenile court observations, mentoring skill instruction, role playing, cultural competencies and awareness of social justice issues, adolescent development instruction, and orientation to systems from which youth were referred.	Throughout the 12 sessions, mentors received ongoing training and supervision by family therapists and graduate trainees. To be flexible and attuned to the needs of mentees, mentors were encouraged to individualize their approach, tailor program activities, and utilize therapeutic staff to troubleshoot problems, obtain support and supervision, and partner on therapeutic interventions as needed (e.g., suicide assessments, engaging with parents).	Mentors were undergraduate students; a little over 10% (12.9%) of mentors were freshman, 28.2% were sophomores, 30.9% were juniors, and 28.0% were seniors in college. Mentors were accepted into the program following an application process and were required to pass a criminal background check. Mentors were mostly female (82.3%) and had a mean age of 20.81 years (SD = 2.94). Most mentors self-identified as White (76.4%), 13.8% as Hispanic/Latino, 2.8% as African American/Black, 2.0% as Asian American, 0.2% as Native Hawaiian/Pacific Islander, and 4.8% as multiracial	Eligibility criteria included: (a) mentees were 11-18-years-old, (b) mentees had experienced at least one risk factor as indicated on the risk assessment (Herrera et al., 2013) at intake, and (c) mentees were available to participate in CC during the program scheduled hours. The current sample included 458 mentor-mentee dyads. Three mentees revoked their consent and thus had missing data on all the study variables. Of the 455 mentees in the study sample, 57% were male with a mean age of 14.3 years (SD = 1.81). A little over half of youth identified as White (56.9%), 27.1% as Hispanic/Latino, 10.5% as multiracial, 3.3% as African American/Black, 1.3% as American Indian/Alaska Native, 0.2% as Asian American, and 0.7% did not report on race/ethnicity	First, using youth- and mentor-report of relationship quality, environmental, but not individual, risk was negatively associated with mentoring relationship quality . . . Second, as expected, mentors, experiences with the program were positively associated with mentoring relationship quality, and in many cases, above and beyond youth level of risk . . . Finally, our hypothesis with respect to the third research question,Äîthat program experience would temper the negative association between youth risk and mentoring relationship quality,Äîwas partially supported. There were no significant moderation effects for the models assessing individual risk and relationship quality, but three of five program experience variables moderated the relationship between environmental risk and relationship quality.

			changed every few weeks and participated with their mentor and additional matches. All activities occurred on site at the University in the presence of other mentor-mentee matches					
Haddock SA, Weiler LM, Lee H, Henry KL, Lucas-Thompson R, Zimmerman TS, et al. Does Organizing Mentor-Mentee Matches into Small Groups Enhance Treatment Effects in a Site-Based Mentoring Program for Adolescents? Results of a Randomized Controlled Trial. Journal of Youth and Adolescence. 2020;49(9):1864-82.	Broadly speaking, the main question was whether mentoring groups were a better method by which youth garnered positive experiences within site based programs. Or, was it sufficient to engage matches in the site-based program independent of such groups? Accordingly, the purpose of this study was to test the impact and process of mentoring groups (i.e., small groups of four mentor-mentee matches) within a site-based	Randomised controlled trial	This 12-week preventive intervention targets at-risk adolescents through an intentional multi-level mentoring community facilitated by experienced mentor coaches and family therapists. Youth are matched one-to-one with an undergraduate university student mentor who is enrolled in a 3-credit service learning course (cf. Masked for review, 2013). The mentoring pairs meet 4 h per week on campus and engage in a semi-structured program including walks on campus, academic support, dinner and prosocial activities.	During training, mentors learn and practice how to facilitate the youth positive interactions with positive developmental setting features. For example, mentors are trained to facilitate their mentees, feelings of belonging and mattering during the initial stages of the program by introducing their mentees to other mentors and mentees. In later stages, mentors are trained to facilitate their mentees, relationship with others in the	[Author note: Not described]	[Author note: Not described]	The sample consisted of 676 mentees: 338 in the treatment condition (i.e., mentoring group) and 338 in the control condition (i.e., no mentoring group). This total sample size represents all mentees who started the program (even if they dropped out later) and excludes mentees who completed the intake process in the months prior to the start of the study but did not begin the intervention. Table 1 presents demographic and background characteristics for the sample of youth. Overall, participants in the treatment and control groups were comparable. The	In this study, better scores on the outcomes of interest were observed at post-intervention (as compared to pre-intervention) for most measured variables in both conditions (mentoring groups and dyad-only). Mentees in both conditions exhibited reductions in behavioral difficulties, anger, anxiety, depression and internalizing behaviors and improvements in conscientiousness, developmental assets, socio-emotional competencies, meaning in life, and self-efficacy. Mentee sense of belonging and mattering in the program (compared to their pre-intervention expectation) also improved for both

	mentoring program.			program through a variety of means, such as sharing a compliment about their mentee with other mentors and staff, connecting their mentee with a mentor who shares a similar or unique life experience, or asking another mentor to provide guidance on a specific career interest or specialized topic of homework. Mentors also learn to promote the mentees, self-efficacy by finding possibilities for their mentee to assume a leadership role (e.g., facilitating a program activity with them or join) or share a talent (e.g., sing during a program activity), and by giving them as many choices as possible within the existing structure of the program (e.g., where to go on the weekly campus walks).			control condition, however, included more White mentees than the treatment condition ($p < 0.05$) and mentees in the treatment condition showed lower academic grades compared to mentees in the control condition ($p < 0.05$). No other differences were found.	conditions. Contrary to what was hypothesized, however, no differences were observed in youth outcomes, mentor relationship quality, or setting quality as a function of treatment condition. In other words, there was no advantage or disadvantage to organizing matches within groups. The assumption that the larger community context within Campus Connections would be inaccessible and ineffective without mentoring groups was not supported in the findings. Indeed, matches were able to glean benefit in spite of not experiencing the smaller group setting.

Stark C, Severn K, Cammell M, Wehrman J, Solis A. Campus Connections Youth Mentoring Program: Cultivating Connection during the COVID-19 Crisis. The Chronicle of Mentoring and Coaching. 2020;1(13):84-9.	The study explored relationships between mentors and diverse youth (ages 10-15), examining changes in mentors, academic achievement, meaningful experiences that contributed to both mentor and mentee socioemotional development, protective factors and strength-based resources, and changes in mentorship processes due to required physical distancing measures in response to COVID-19.	Qualitative research	The University of Colorado Colorado Springs (UCCS) Campus Connections (CC) youth mentoring program utilizes the Mentor Model (Weiler et al., 2014) to match at-promise youth from historically oppressed backgrounds with undergraduate student mentors. The CC program facilitates relationships between undergraduate student mentors and diverse youth (ages 10-15) to cultivate a network of resources and promote academic success for both mentors and mentees, provide meaningful experiences that contribute to socioemotional development, and increase protective factors and overall resiliency . . . Campus Connections is further distinguished from relationship-focused mentorship models as it utilizes counselors-in-training (under the supervision of university faculty) as therapeutic support for both mentors and mentees. The primary role of these counselors is to utilize listening and responding skills while providing brief therapeutic interactions with youth, employ the PYD perspective, and support mentors as they engage in ethical decision-making (Krafchick et al., 2019).	Mentors participate in a CC practicum course that provides guidance and support during experiential and service learning (Bringle & Hatcher, 1995) activities . . . All CC counselors and mentors receive onboarding training, and ongoing education and support during the CC service-learning practicum course and post lab meetings after every CC session. The PYD perspective is infused into all curriculum, training, course meetings, and interactions between youth, mentors, and CC staff.	Campus Connections is further distinguished from relationship-focused mentorship models as it utilizes counselors-in-training (under the supervision of university faculty) as therapeutic support for both mentors and mentees.	[Author note: 7 mentors, 71% female, 29% male, 43% White, 71% 18-24 y.o.]	age 10-15	The preliminary results from this study confirm the themes identified by Weiler et al. (2014) and Haddock et al. (2013), while also revealing themes regarding influence of the diversity of mentor and mentee identities on the mentorship process, as well as the impact of required transition to a virtual environment due to the COVID-19 response. Mentors described themselves as having a variety of identities that lead to experiences of discrimination within institutions (particularly the university) and the greater community. All mentors reported instances where they were able to empathize with the youth based on their disadvantaged identities and adverse life experiences. They further describe these instances of increased empathy as supporting the building of rapport. The rapport between each mentor and their mentee, their mentor-family, mentor-coach, CC practicum course instructor and CC staff was consistently referenced as the foundation of support that enabled the mentor to adapt and achieve self-growth. Rapport within CC was the common factor described by all mentors as the source of resilience, support and strength that enabled them to engage in the following actions: Engage with the mentee in the virtual environment; identify challenges and improvise and adapt in the moment; engage in

									exploratory dialogue with supportive persons in CC; participate in self reflection, self-care, and self-growth; implement new strategies for addressing challenges and improving interactions with mentee. The new structure and schedule resulted in less one-on-one time between mentors and mentees. One mentor participant described this decrease in time spent with his mentee as challenging his ability to bond: We were able to create a larger bond and able to help [them] develop and grow within those five hours per week . . . transitioning online we had one hour per week . . . [we] had to adapt how you might connect. Mentors described a variety of challenges associated with the transition to remote interactions with their mentees. Each participating mentor described at least one particularly meaningful interaction with a mentor-coach, member of their mentor family, or CC instructor or staff regarding challenges faced during the transition to remote mentoring. Upon consulting with supports within CC regarding the challenges associated with remote mentoring, mentors described themselves as engaging in self-reflection regarding their own responses to the changes due to the COVID-19 pandemic.

Stark C, Wehrman J, Stutey D, Solis A, Severn K, Cammell M, et al. Therapeutic Mentoring for Multiply Marginalized Y outh: A Grounded Theory Study of Mentors' Adaptation During the COVID-19 Crisis. Journal of Human Services. 2021;Fall:116-32.	We used Grounded Theory Methods (GTM) to explore the unique experience of mentors in the CC program as they adapted to challenges and changes associated with the COVID-19 pandemic.	Qualitative research	Campus Connections (CC) is a therapeutic youth mentoring program that exemplifies a sustainable approach to providing at-promise youth with mentoring services while simultaneously providing therapeutic support. This creative university-based mentoring program targets multiply marginalized youth (ages 10-18), prioritizing the inclusion of youth from communities who experience social inequity and oppression: Lesbian, Gay, Bisexual, Transgender, Queer, plus (LGBTQ+) youth; those in the juvenile justice system; and youth who experience socioeconomic disadvantage. While participating in CC, youth are paired one-on-one with highly trained undergraduate student mentors from a variety of university disciplines. Mentor-mentee pairs are assigned a counselor who is a graduate student in the master in counseling program; the counselors provide direct therapeutic services to youth mentees and their families, and support mentors in navigating the mentor-mentee relationship. Each youth cohort meets for 12 weeks per semester, engaging in weekly 4-hour structured sessions with activities that aim to enhance connection with others and community, foster social-emotional growth, increase study skills and other academic abilities, and cultivate positive identity development (Krafchick et al., 2019).<br><br>Youth participants are described as multiply marginalized youth who can thrive when appropriate resources are accessible (Cyrus, 2017) and changes occur in institutional structures that create and maintain inequality (Swadener & Lubeck, 1995, p. 3). CC youth	As part of their participation in CC, mentors are enrolled in a 3-credit hour 16-week fieldwork course. Mentors receive foundational training during this course, with community members and program faculty facilitating trainings in trauma, violence, and resiliency; culturally responsive practice; implementing reflective listening skills; and boundaries and ethics.	While engaging in CC programming, mentors also complete weekly reflective journal entries.	Mentors who participated in the study were enrolled in the undergraduate Fieldwork in Human Services course at a mid-sized university in the midwestern United States. In fulfillment of the requirements for the course, mentors attended each CC session, completed course assignments and activities, and served as a mentor for their assigned youth mentee for 12 weeks. A total of 22 undergraduate students were enrolled in the course and served as mentors during the Spring 2020 semester. The faculty member teaching the fieldwork course invited all mentors to participate in this study, and 10 of the 22 mentors (45%) consented to participate. Of those mentors who consented to participate in the study, five (50%) described their ethnicity as Black/African American, Native American, Hispanic/Latino, or Asian. Although the demographic questionnaire did not inquire about mentors, sexual orientation, one mentor (10%) voluntarily disclosed during the semi-structured interview that they are queer and are a member of the LGBTQ+ community. Table 1 summarizes the demographic	[Author note: Not described]	Results and emerging themes highlight the importance of adaptation as a consistent driver of change throughout the experience. In line with current research, the transition to conducting mentorship activities in the virtual setting is associated with several challenges, from difficulty connecting emotionally in virtual environments, to limited time and space for private discussions with mentees (Ettekal & Agans, 2020). These challenges required mentors to adapt their approach, learning new ways to connect with their mentee, while also working through their own emotional response to the COVID-19 crisis.

			participants are predominantly members of historically oppressed communities who continue to experience multiple intersections of oppression and discrimination. These youth are often labeled at-risk due to mental health symptoms or difficulty persisting in certain environments. Referencing these youth as multiply marginalized acknowledges that these difficulties can be attributed to systemic factors rather than individual pathology.<br><br>To address these systemic variables, CC faculty and counselors help mentors toidentify areas where systemic change is needed, and support mentors, advocacy efforts to effect change in youth school and/or community environments. In addition to these advocacy efforts, counselors facilitate discussion with youth and mentors during social justice focused activities aimed at enhancing understanding of the role of privilege and oppression in access to resources, and day-to-day experience (Krafchick et al., 2019).			information for the mentors who participated in this study. Multiple mentors described themselves as first-generation and non-traditional college students. Those students who described themselves as non-traditional shared challenges to adapting to the virtual environment due to difficulties learning to use technology and struggles to fit in with their peers		

Stark C, Estrada S, Rockwell E, Severn K, Burr B, McTernan L, et al. Campus Connections Youth Mentoring Program: Lessons in Ethical Development. The Chronicle of Mentoring and Coaching. 2022;6(15):502-9.	This study aims to address the following research question: How do undergraduate student mentors, experience the facilitation of youth-focused ethics curriculum, implemented by graduate assistants with mentors and their youth mentees during the 12 week 12-week CC youth mentoring program?	Qualitative research	In spring of 2022, CC therapeutic youth mentoring program implemented the DFEI Finding your ethical compass LAP lessons (Stark et al., 2022). Originally created for high school students, these lessons were newly adapted for use with middle school-age youth and implemented for the first time as part of this study. Campus Connections prioritizes the inclusion of multiply marginalized youth (Cyrus, 2017) from local suburban and rural communities. Over the 12-week program, highly trained undergraduate student mentors meet weekly with their youth mentee (age 10-14 years old) and are engaged in ethical decision-making exercises. Each exercise supported social and emotional learning through scaffolded interactions and group processing (Stark et al., 2022).	...highly trained...	[Author note: Not described]	Four of the 17 mentors participating in CC consented to participate in the study.	[Author note: Not described]	The four mentors interviewed for this study described their process of ethical development and the role of their lens in their perceptions and interpretations. Mentor participants described their processes for identifying ethical dilemmas and engaging in ethical decision-making. The core ethical lessons learned from this process are deemed ethical humility, which include mentors, ability to acknowledge their own fallibility, appreciate the views and strengths of others, and cultivate ethical perspective that transcend self-interest.

Stutey DM, Solis AE, Severn K, Notestine L, Enkler KL, Wehrman J, et al. Perceptions of At-Promise Youth in a Therapeutic Youth Mentoring Program. The Professional Counselor. 2022;12(1):17-35.	The purpose of this study was to examine and gain further insight into participants, lived experiences with a therapeutic youth mentoring program, specifically, a therapeutic youth mentoring program in which the mentors had additional training and supervision in mental health areas and additional support was provided by CITs while under supervision. The overarching question for this study was: What were participants, lived experiences prior to and after participating in a therapeutic youth mentoring program?	Qualitative research	All youth participated in the 12-week CC therapeutic youth mentoring program that met on the university campus, once a week for 4 hours. This therapeutic youth mentoring program was designed to bring youth to a college campus to help them become more comfortable and familiar with future college and career goals. Youth followed a schedule that consisted of: working for 60 minutes with their mentor on academic and study skills, going on a 30-minute walk and talk to learn about different locations on the university campus each week, participating in a 30-minute family-style meal at the dining hall with their mentor family, choosing two 45-minute prosocial or social justice activities.	All mentors attended an orientation and background screening prior to being accepted for the program. The mentors who were selected spent the first 3 weeks of the semester in intensive training with counselor educators, who were also licensed professional counselors, to learn about child abuse reporting, ethics of working with minors, crises and trauma, and basic counseling skills.	All CITs were in their last semester of graduate studies and met weekly with counselor educators for supervision. Two counselor educators and supervisors, also licensed professional counselors, were also present each evening of CC to support mentees, mentors, and CITs.	CC mentors were recruited and selected from a variety of undergraduate disciplines across campus, with more than half being from counseling and human services, psychology, or education majors . . . Each mentor family consisted of approximately three mentor/mentee pairs. In this study, there were a total of three mentor families and each family was assigned a mentor coach. The mentor coaches were graduate-level counseling students who provided their mentor family with support throughout the semester. In addition, four graduate-level CITs were selected to provide ongoing counseling to mentees throughout the therapeutic mentoring program. All CITs were in their last semester of graduate studies.	Of the 18 youth enrolled in CC, 14 youth agreed to participate in the research study. The participants in this study consisted of 14 youth, ages 11-15, with the following demographics: 71% male, 21% female, and 7% transgender/gender-expansive; 57% White, 29% Black/African American, 21% Hispanic/Latino, 14% American Indian/Alaska Native, and 7% Asian; and approximately half on free and reduced lunch. School district partners were asked to consider and recommend at-promise youth if they were not reaching their full potential and might be vulnerable to school dropout, substance use/misuse, and/or criminal behavior.	. . . the participants in this study shared that having a mentor had a positive impact on their overall behavior (DuBois et al., 2011; Tolan et al., 2014; Weiler et al., 2015). Specifically, participants indicated more adaptive and resilient thinking after participating in the youth mentoring program . . . Being able to walk away from a fight or potentially violent situation and asking adults for help were two of the outcomes participants described. A few participants even cited the therapeutic youth mentoring program as the reason why they have developed better coping strategies to better handle the stress they feel in their daily lives. Another finding consistent with the research on mentoring programs was that participants acknowledged the significance of the primary mentoring relationship (Weiler et al., 2015; Weiss et al., 2019). One significant finding between the initial and final interview was the participants, shared perspective that they trusted their mentor and the other adults supporting the youth mentoring program (e.g., mentor coaches and counselors) . . . Many participants also described that because of the trust they developed with their mentor, they were able to also trust other adults in their lives, specifically parents, counselors, and teachers . . . Although the majority of the participants shared

								the positive impact of having a therapeutic component to this youth mentoring program, five of the 14 participants still shared in the final interview that they were hesitant to meet with a counselor (either at school or at the therapeutic mentoring program). Haddock and colleagues (2017) posited that youth who participated in CC might be embarrassed to share about their experiences with counselors. It is important to note that some of the youth participants also did not make the distinction between CITs and mentor coaches. So, more clarity on the role of CITs in youth mentoring programs may be needed.

Rempe G, Saltis M, Matheson D, Cople S. Norm-Referenced Effects of a Campus-Based Therapeutic Mentoring Program. Journal of Youth Development. 2023;18(2).	The purpose of this study was to track the emotional and behavioral functioning of mentees over the course of the CC program as measured by a validated and change-sensitive behavioral monitoring tool, and to explore the potential moderating influence of mentees, caregiver-identified sex assigned at birth. This study specifically examined changes in mentees, self-reported conduct issues, mood, cognition, social skills, and academic functioning over time using the Behavior Intervention Measurement Assessment System, Second Edition.	Single group study	Campus Connections (CC) is a community-based mentoring program for youth that was designed with recent researched-based recommendations in mind (Eccles & Gootman, 2002; Haddock et al., 2013, 2020; Kelly et al., 2000; Rhodes, 2005; Tseng & Seidman, 2007; Weiler et al., 2015, 2019). This program was designed as a community-engaged learning opportunity for enrolled college students to serve as mentors over the course of an academic semester, who work with youth directly in mentoring dyads within a group setting. CC specifically expands beyond the traditional mentor-mentee dyad to include grouping of mentor-mentee families while also introducing mentees to numerous other adults and services on a university campus (e.g., undergraduate/graduate students, community partners, therapists) to expand their social networks, as recommended by Schwartz and Rhodes (2016). Community partners who referred youth to the program, such as personal counselors, school officials, and probation officers, are updated on the weekly progress of mentees to ensure collaboration and communication with various stakeholders. The group format also includes a celebratory graduation ceremony at the end of each semester in which parents and community supporters of the mentees are invited to celebrate their progress and successes within the program. The mentor dyads are chosen individually by the mentees, who hand-select their mentor based on a collage of the mentor interests and hobbies. CC spans 16 weeks during fall and spring academic semesters, consisting of four weeks of mentor training and 12 weeks working directly with	There is also a four-week training period within the program during which mentors are introduced to basic relational skills, diversity factors, and key findings from the mentoring literature to further their understanding of the mentoring process. Training immediately precedes mentee participation in the program.	Mentors and mentees are grouped into mentor families that are supervised by mentor coaches who have experience and additional training within CC and the helping professions. Mentor coaches are supervised by doctoral-level graduate students or instructors who hold a professional license or are license-eligible in counseling. Instructors are further supervised by university faculty from the departments of Counseling Psychology and Counselor Education and Supervision.	College students	Participants included 52 mentees between 11 and 17 years of age who were enrolled in a CC program that was implemented in a medium-sized university within the Rocky Mountain region. The sample for this study was collected over two consecutive semesters during which the program was implemented; fall 2017 (N=25) and spring 2018 (N=27). Based on caregiver-reported sex assigned at birth, the combined sample was 56% male. A small number (N=5) of participants participated in both semesters of the program.	Primary findings indicated that youth participation in CC was associated with improvement on all BIMAS-2 subscales (i.e., conduct, negative affect, cognitive/attention, social, academic functioning).

			mentees. This results in approximately 48 hours of direct mentoring with youth. Mentor pairs meet for four hours each night, which includes walk and talks, individual tutoring provided by mentors (i.e., academic support time), sharing a meal, and two hours of prosocial programming and activities each week. The goals of CC include providing prosocial skill development, supporting career and academic success, and promoting individual growth for youth who are typically referred by community organizations (e.g., probation, court, the department of human services, pretrial services, public schools, programs for youth with disabilities). CC expands upon the traditional dyad model proposed by Rhodes (2005) and takes a developmental approach to mentoring (Karcher et al., 2002) by facilitating bidirectional social-emotional growth in a structured environment and encouraging mentees and mentors to grow into expanded roles within the program as they progress. CC further focuses on fostering rich connections beyond the mentoring dyad as recommended by Schwartz and Rhodes (2016), attempts to build upon mentees, adaptive factors (Sieving et al., 2017), and adds a layer of therapeutic support through the availability of mental health professionals.						

Lucas-Thompson RG, Miller RL, Moran MJ, Rzonca A, Krause JT, Montavon JK, et al. “Scaling Out” a Mindfulness-Based Intervention Through a Youth Mentoring Program: Preliminary Evidence for Feasibility, Acceptability, and Efficacy. Mindfulness. 2024;15(4):872-88.	Our goal was to explore the feasibility and acceptability of scaling out of an MBI through a mentoring program that targets adolescents facing multiple adversities, with the long-term goal of increasing the reach of evidence-based MBI to reduce mental health symptoms in adolescence. We conducted a randomized feasibility study comparing mentoring alone to mentoring plus MBI; to examine the feasibility of this scaling out strategy, we compared attendance and acceptability ratings between those randomly assigned to the two different conditions. In addition, to examine the possible benefits of improving regulation through teaching mindfulness, we compared change in important and relevant outcomes (emotion regulation, attention problems, externalizing behaviors, internalizing behaviors, posttraumatic stress disorder symptoms) between those who	Randomised controlled trial	In the current study, we partnered with a time-structured (12-week), formal mentoring program called Campus Connections Therapeutic Youth Mentoring (CC) (Haddock et al., 2020; Weiler et al., 2015). CC takes place on a university campus. The program was developed in response to community needs for youth services and is therefore strongly supported by local schools, the juvenile justice system, and the state. Youth ages 10-18 who have experienced multiple adversities are referred to CC. University students serve as mentors, and receive extensive and ongoing training throughout the program including how to facilitate high-quality mentoring relationships. Specific activities in the program support both the establishment of mutual, trusting, and empathetic mentoring relationships and specific prevention-focused activities (e.g., supporting academic success through a tutoring-like experience). CC has high rates of attendance and retention, serves youth facing multiple adversities, and is related to reductions in problem behaviors as well as gains in healthy identity dimensions (e.g., future orientation), social-emotional competencies, and decreases in internalizing symptoms (Haddock et al., 2020; Weiler et al., 2015).<br><br>Across both semesters, mentor/mentee pairs who were randomized to receive mentoring plus MBI participated in the manualized mindfulness program, L2B, led by trained graduate student facilitators and supervised by the first author. Each L2B session consisted of exercises and group discussions focused on one of the following themes:	Prior to the start of the CC semester, undergraduate students enrolled in the CC service learning course completed 18 h of mentor training and created mentor profiles with self-reported characteristics and interests.	[Author note: Not described]	Of the mentors who provided information on their gender, 88.9% were female, 8.1% were male, and 3% selected another gender identity. Of those who provided their racial/ethnic background, mentors were 71.7% White, 9.1% multiracial, 6.1% Hispanic/Latino, 4% Asian, and 2% Black. Approximately 7.1% of mentors did not provide this information.	Participants were youth enrolled as mentees in CC for one semester (n=99). Adolescents who had experienced multiple adversities were referred by schools and community agencies (e.g., Department of Human Services involvement, juvenile justice involvement, behavioral and/or emotional concerns). At baseline, 95% of adolescents had experienced at least one risk factor (M=4.57, SD=2.54, range 0-10) on the baseline risk assessment (Herrera et al., 2013), which assessed environmental (e.g., economic adversity, family risk/stress, and peer difficulties) and individual (e.g., problem behaviors and mental/health concerns) risk factors. On average, mentees were 14 years old (SD =2.19; range, 10-19 years old). The slight majority of mentees were male (53.5% of those who provided their gender) and non-Hispanic White (54.5% of those who provided their race/ethnicity). However, 37.4% were female, 4% were transgender, and 5.1% chose another in regard to their gender identity. In terms of race/ethnicity, remaining participants were 27% Hispanic/Latino, 6% Native American, and 5% Asian/Pacific Islander or Black, and 7% self-identified as	The goal of the current study was to provide an initial examination of the feasibility, acceptability, and potential efficacy of scaling out an MBI through a mentoring program targeting adolescents facing multiple adversities, through a randomized feasibility trial comparing mentoring alone to mentoring plus mindfulness. Results indicated that the addition of an MBI to the mentoring program did not affect attendance, but did relate to small increases in overall program acceptability. In addition, analyses focusing on condition differences in change in psychological outcomes over time indicated that adolescents who received mentoring plus MBI demonstrated more favorable change in emotional clarity and in controlling impulsive behaviors when distressed (two dimensions of emotion regulation), as well as greater decreases in attention problems, externalizing symptoms, and PTSD symptoms. In contrast, adolescents who received mentoring alone demonstrated more favorable change in perceived accessing of effective emotion regulation strategies (another dimension of emotion regulation) and internalizing symptoms. Many but not all of these effects were more pronounced when focusing specifically on older, high school-aged adolescents, relative to effects observed in the

	did and did not receive mindfulness training through the mentoring program.		awareness of body, awareness of thoughts, awareness of feelings, stress management, compassion for self and others, and healthy habits of mind. Notably, in the semester that L2B was provided virtually, MBI sessions were slightly shorter than those provided in-person, though the content and activities of the MBI were largely unchanged.				holding two or more racial identities. Parents of mentees reported that the median household income ranged from \$40,000 to \$59,999, and an average education level between having completed some high school and having a high school diploma.	full sample of 10-18-year-olds.

Miner-Romanoff K, Greenawalt J. Evaluation of the Arthur Project: Evidence-Based Mentoring in a Social Work Framework with a Social Justice Approach. Societies. 2024;14(7).	Does structured, intensive in-school therapeutic mentoring with social work interns increase the socioemotional, cognitive, noncognitive, and academic skills and engagement of underserved middle-school students? A mixed method approach, with quantitative surveys and qualitative focus groups, was deemed best to capture the TAP outcomes as comprehensively as possible and provide increased validity. Quantitative surveys elicited participants, self-assessments in many mentored areas. Focus groups prompted subjective, in-depth responses from participants and other key stakeholders	Mixed methods	TAP seven goals encompass the range of academic, mental, emotional, and social factors that warrant development in most underserved middle-school youth.<br>1. Cultivate youths, social-emotional wellness and mental health.<br>2. Increase academic engagement.<br>3. Provide youth with leadership, goal achievement, problem-solving, knowledge, self-advocacy, and communication skills to reach their full potential.<br>4. Provide the skills to successfully transition to high school and beyond.<br>5. Foster family engagement.<br>6. Encourage culturally responsive community and civic engagement.<br>7. Apply an antiracist, social justice lens to mentoring in furtherance of awareness and attunement to wider social impacts and social change.<br><br>TAP implemented the mentor program with 200 students in 6th through 8th grades in two schools in The Bronx and Brooklyn in New York City. Each of the 30 mentors (social work interns) was matched with 4-10 mentees. Mentors and mentees can spend up to 500 h in focused programming, far exceeding most mentoring programs [21]. Mentors provide services to students through individual school-based counseling sessions, small afterschool group work, and Saturday community-based activities. As part of the program, TAP also supports a group of parents and guardians. Consistent with TAP focus on healing and liberation, multiple program components, events, and goals are based on the framework of French et al. [22] for radical healing in communities of color. These include collectivism, critical	TAP mentors are trained in healing-centered engagement; this is an alternative approach to trauma-informed care. Consistent with best practices, all mentors receive initial and ongoing weekly training related to mentoring. The training includes relevant research studies to bridge theory and relate it to therapeutic mentoring. Furthermore, aligned with TAP value of working toward equality and equal justice for all, mentors participate in social justice training modules aligned with critical mentoring, the understanding of social justice issues that impact the mentees, lives. Finally, compatible with best practices, TAP provides mentors crisis training and support throughout the entire period	The mentors are social work clinicians-in-training who have chosen to apply their internship clinical training hours to service in the TAP program and are supervised by university and external supervisors and the TAP Executive Director. [LCSW & doctorate in social work and sociology]<br><br>For every mentee, mentors are required to submit quarterly reports that include individualized growth plans, biopsychosocial assessments, and progress notes. At the end of each year, mentors also write closure letters to their mentees and letters to the next mentor class	The mentors are social work clinicians-in-training who have chosen to apply their internship clinical training hours to service in the TAP program and are supervised by university and external supervisors and the TAP Executive Director. Screening and interviewing of the mentors takes place for their knowledge of therapeutic mentoring and social justice. Approximately 95% of the mentors are of minority ethnicities.	Within the qualifying schools, 6th-, 7th-, and 8th-grade students are generally referred to TAP by the schools based upon a pattern of absences, poor grades, negative behavioral challenges, chronic absenteeism, and social-emotional needs. Referrals may come from the youth, the youth family, or school staff. All youth voluntarily participate in the program. Approximately 95% of the mentees were people of color.	With regard to anxiety levels, pre- and post-test results indicated no large differences, although the scores in both cases indicated high anxiety. Depression scores were higher post-program than pre-program, as were traumatic stressors. The scores point to the need for ongoing counseling for these youths. In addition, several mentors reported that their mentees seemed more comfortable and responsive with the assessment at the end of the year. These observations may indicate that the mentees had greater confidence in themselves and this was communicated more openly after TAP. With regard to social-emotional growth, student surveys in all three grades showed growth in persistence, perseverance, and confidence (Table 2), and 7th- and 8th-graders, self-advocacy and self-help (Figure 1). For cognitive skills growth, teachers observed students, improvement in help-seeking (Figure 2) and 6th-grade students reported increased mentor help in setting goals (Figure 7) and in reaching goals (Figure 8). Seventh and eighth graders recognized that their mentors helped them accomplish many things (Figure 9). It would appear that skilled one-to-one mentoring greatly aids these youths gain confidence and persistence in succeeding in school and preparing for the future. With regard to greater academic engagement,

			consciousness, racial hope, strength and resistance, and cultural authenticity and self-knowledge. In 2023, TAP also introduced healing-centered engagement as an anti-oppression alternative to trauma-informed care [23]<br><br>Mentors and mentees meet individually during the school day 1-2 h weekly. They also meet in small mentor families 2 to 4 h weekly. Mentors participate too with the students in the Saturday activities that further a sense of community and provide experiential opportunities.<br><br>The Arthur Project has adopted many of its philosophies and structured programming elements from therapeutic mentoring. Creating individualized growth plans and in recognition of the youths, stated goals, the mentors help them develop their communication skills, conflict resolution abilities, and confidence-building [25]. Mentees and mentors are matched during group matching social and networking events [26]. Responding to the mentees, requests, TAP also instituted group mentoring. The groups can decrease mentees, feelings of isolation, increase their confidence and commitment, and<br>provide networks of multiple role models [27]. Mentors are encouraged to advocate for their mentees in, for example, maintaining contact with caregivers, ensuring mentees, access to services, introducing them to contacts and role models, and vouching for them in court proceedings. Advocacy can help mentees, development and self-confidence and widen social networks that may lead to promising relationships [28].					the results were similar. Mentees,, teachers,, and caregivers, surveys and qualitative results indicated TAP increased mentees, engagement.<br><br>Based on the quantitative and qualitative results, the TAP program of 2022-2023 was highly successful. Goals 1, 2, 3, and 4, social-emotional wellness; academic engagement; leadership, problem-solving, self-advocacy, communication skills; skills for high school transition,Äîwere met very successfully. However, mental health was not measurably improved as a result of TAP participation. More mentees reported depression and PTSD symptoms after the program. Based on their greater sharing after the program, it can be assumed that their bonds and trust had grown and become solidified. Perhaps the end-of- year assessment was more accurate than that at the start. Nevertheless, the depression and PTSD symptoms warrant closer examination
